# Advances in Selective Electrochemical Oxidation of 5‐Hydroxymethylfurfural to Produce High‐Value Chemicals

**DOI:** 10.1002/advs.202205540

**Published:** 2022-12-08

**Authors:** Lei Guo, Xiaoxue Zhang, Li Gan, Lun Pan, Chengxiang Shi, Zhen‐Feng Huang, Xiangwen Zhang, Ji‐Jun Zou

**Affiliations:** ^1^ Key Laboratory for Green Chemical Technology of the Ministry of Education School of Chemical Engineering and Technology Tianjin University Tianjin 300072 China; ^2^ Collaborative Innovative Center of Chemical Science and Engineering (Tianjin) Tianjin 300072 China; ^3^ Zhejiang Institute of Tianjin University Ningbo Zhejiang 315201 China; ^4^ Haihe Laboratory of Sustainable Chemical Transformations Tianjin 300192 China

**Keywords:** biomass upgrading, electrocatalyst, HMF oxidation electrolysis, reaction mechanism, reactor

## Abstract

The conversion of biomass is a favorable alternative to the fossil energy route to solve the energy crisis and environmental pollution. As one of the most versatile platform compounds, 5‐hydroxymethylfural (HMF) can be transformed to various value‐added chemicals via electrolysis combining with renewable energy. Here, the recent advances in electrochemical oxidation of HMF, from reaction mechanism to reactor design are reviewed. First, the reaction mechanism and pathway are summarized systematically. Second, the parameters easy to be ignored are emphasized and discussed. Then, the electrocatalysts are reviewed comprehensively for different products and the reactors are introduced. Finally, future efforts on exploring reaction mechanism, electrocatalysts, and reactor are prospected. This review provides a deeper understanding of mechanism for electrochemical oxidation of HMF, the design of electrocatalyst and reactor, which is expected to promote the economical and efficient electrochemical conversion of biomass for industrial applications.

## Graphical abstract

This review provides a deeper understanding of mechanism for electrochemical oxidation of 5‐hydroxymethylfurfural (HMF), the design of electrocatalyst and reactor, and points out the possible important development orientation, which is expected to promote the economical and efficient electrochemical conversion of biomass for industrial applications.

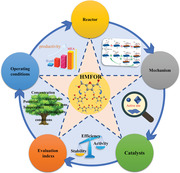

## 1 Introduction

With the rapid development of the global economy, human demand for energy and chemicals is increasing, which is heavily dependent on the utilization and conversion of fossil fuels, resulting in energy crisis and environmental issues.^[^ ^1^ ^]^ Therefore, it is urgent to seek sustainable alternatives to petrochemical products. Because of the renewable and abundant reserves, biomass is expected to solve the sustainable production of high value‐added chemicals and fuels.^[^ ^2^ ^]^ Lignocellulosic biomass is considered as the most abundant and bio‐renewable carbon‐neutral resource with a wide geographical distribution, which has received intensive attention over the past decade.^[^ ^3^ ^]^

**Scheme 1:**
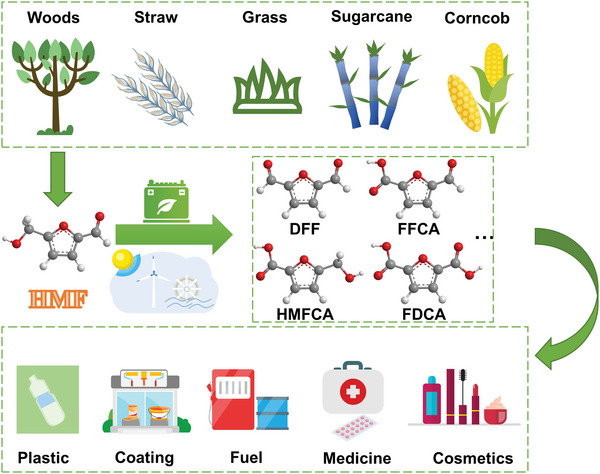
Schematic diagram of green transformation for HMF and the wide application of value‐added chemicals.

5‐hydroxymethylfural (HMF), which can be obtained from lignocellulose biomass or fructose in an environment‐friendly way,^[^ ^4^ ^]^ is rated as an important bio‐based C_6_ platform molecule.^[^ ^5^ ^]^ Because of the special structure that hydroxymethyl and formyl coexist on the furan ring, HMF has become a multifunctional molecule which can be oxidized into chemicals with higher added value (**Scheme** **1**).^[^ ^6^ ^]^ The product of 2,5‐diformyl furan (DFF), 5‐hydroxymethyl‐2‐furancarboxylic acid (HMFCA), 5‐formyl‐2‐furancarboxylic acid (FFCA), and 2,5‐furandicarboxylic acid (FDCA) can be obtained, and each of them has wide application prospect. For example, HMFCA is a valuable monomer in polyester synthesis and FFCA has broad application prospects in fuel, chemical intermediates, drugs, and so on.^[^ ^7^ ^]^ DFF is an important raw material in the synthesis of pharmaceutical intermediates, diamines, antifungal agents, polyols, and polyethylene.^[^ ^8^ ^]^ In addition, FDCA, listed as one of the most promising bio‐derived platform molecules by the U.S. Department of Energy in 2004, is an important precursor for the production of polyethylene furandicarboxylate (PEF).^[^ ^9^ ^]^ It is worth noting that PET is one of the most produced plastics, and its refractory problem can be solved by using bio‐based PEF as a substitute.^[^ ^10^ ^]^

The initial research on HMF oxidation mainly focused on thermal catalysis, where elevated temperature (above 100 °C), high pressure (10 bar O_2_), and noble metals (Au, Pt, Ru, and Pd) are often required to obtain satisfactory yield.^[^ ^11^ ^]^ In addition, organic solvents and oxidants are often involved, which is not in line with the concept of green production. Therefore, there is an urgent need to find a scheme for green transformation of HMF, which is environmental‐friendly with low energy consumption and low‐cost catalyst.^[^ ^12^ ^]^ Among the environment‐friendly chemical upgrading methods, such as photocatalysis and biocatalysis, electrochemical oxidation of HMF has been widely studied in recent 5 years due to its satisfactory efficiency.^[^ ^13^ ^]^ In addition, HMF electrolysis has the following advantages and is worthy of further popularization and application: 1) Aqueous solution can be used as oxygen source to replace high‐cost pressurized gas;^[^ ^14^ ^]^ 2) noble metals can be completely replaced, because various nonnoble metal catalysts can achieve high reactant yield currently;^[^ ^15^ ^]^ 3) product yield can be controlled by the regulation of electrochemical‐related parameters such as current, voltage, electrolyte, and electrocatalyst properties; 4) value‐added products can be generated on both sides of the electrochemical reactor, improving the overall efficiency of the system.^[^ ^16^ ^]^ In addition, the desired pairing reaction can be implemented to achieve flexible production, and the modular design of electrochemical reactor can promote its large‐scale integration;^[^ ^17^ ^]^ 5) as the cost of renewable energy power generation continues to decline, the competitiveness of electrocatalysis will increase day by day.^[^ ^18^ ^]^

At present, the electrochemical oxidation of HMF is often paired with cathode hydrogen production in most studies, which solves the slow reaction kinetics of anode in traditional water electrolysis and improves the energy efficiency. Besides, the risk of crossing hydrogen and oxygen in water splitting can be avoided, and high value‐added chemicals rather than low value oxygen can be obtained. However, the researches mainly focus on the design of high efficiency catalyst, and there is little in‐depth research on the reaction mechanism. Therefore, theoretical guidance is so lacking that cannot help guide the experiment. In addition, there is still a lack of research on the reactor design for HMF electrolysis. From the above point of view, it is necessary to systematically review and discuss the electrochemical oxidation of HMF, from mechanism to reactor design, in order to apply it in industry faster.

There is no unified mechanism about the electrochemical oxidation of HMF so far, thus we first comprehensively summarize the related mechanism and put forward the corresponding opinions. In addition, the standardization of basic research on HMF transformation needs to be established urgently. In view of this, the review then lists many important parameters affecting the reaction performance in order to remind readers of the easily overlooked points in the experiment, hoping to make the future research data worthy of comparison. For designing catalysts with high activity and selectivity, this review expounds the corresponding catalysts in detail according to the different reaction products, combining with the reaction mechanism and active sites. Finally, reactors for HMF continuous electrolysis are introduced, especially membrane electrode assembly (MEA) electrolyzer, which has important guiding significance for actual production although it has not been widely used. The possible problems in actual operation are analyzed in detail such as membrane, porous transport layer, and bipolar plates, and the possible solutions are discussed. In general, this review introduces and discusses the electrochemical oxidation of HMF comprehensively, from mechanism to application, and provides readers with a clearer and broader vision for related research.

## 2 Pathway and Mechanism for Electrochemical Oxidation of HMF

**Figure 1:**
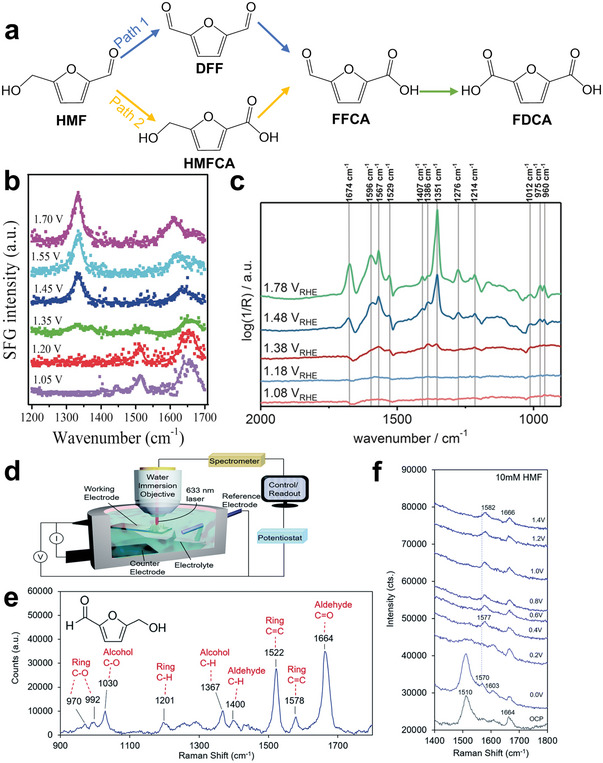
a) Two reaction paths of electrooxidation of HMF; b) SFG spectra with ssp (polarization direction of the light source) polarizations recorded at the working electrode/electrolyte interface after running the cell at various voltages for 90 min. Reproduced with permission.^[^ ^20a^ ^]^ Copyright 2019, Wiley‐VCH. c) Operando ATR‐Fourier transform infrared spectra recorded at various applied potentials between 0.98 V versus reversible hydrogen electrode (RHE) and 1.78 V versus RHE after 20 min of applied potential. Reproduced with permission.^[^ ^21a^ ^]^ Copyright 2018, Wiley‐VCH. d) Operando SERS with a custom‐built reaction cell and water immersion objective; e) a spectrum of a 500 × 10^−3^ m aqueous solution of HMF; f) in situ Raman spectra of HMF under different voltages in 10 × 10^−3^ m KOH. Reproduced with permission.^[^ ^22^ ^]^ Copyright 2020, Royal Society of Chemistry.

There are two main pathways to oxidize HMF to FDCA under a certain bias, involving the preferential oxidation of hydroxyl and aldehyde group, respectively (**Figure** **1**a). Path 1 is that HMF is preferentially oxidized to DFF, and then further oxidized to FFCA. Path 2 is that HMF is preferentially oxidized to HMFCA then oxidized to FFCA. Finally, FDCA will be obtained by deeply oxidization from FFCA at higher potential. Considering that these two pathways may occur at the same time, researchers initially detected the formation of intermediate products by high performance liquid chromatography (HPLC). However, the ex situ characterization cannot directly and accurately reflect the reaction process. Furthermore, some in situ/operando characterizations were used to confirm the pending issues. The results show that the electrooxidation of HMF tends to follow path 1 in nonstrong alkaline environment (pH < 13) while path 2 in strong alkaline environment (pH ≥ 13).^[^ ^19^ ^]^ In detail, only the peaks of intermediate product of HMFCA were detected by in situ sum frequency generation (SFG) vibrational spectroscopy at 1335 and 1380 cm^−1^ in 1.0 m KOH at 1.35 V, while DFF showed no observable signal from the 1300 to 1600 cm^−1^ region (Figure 1b).^[^ ^20^ ^]^ In addition, operando electrochemistry‐coupled attenuated total reflection infrared (EC‐ATR‐IR) spectroscopy detected HMFCA bands at 1386 and 1351 cm^−1^ in 1.0 m KOH at 1.38 V (Figure 1c).^[^ ^21^ ^]^ On the other hand, it was observed that DFF is the product of HMF oxidization under weak alkali environment (pH = 12) with voltage higher than 1.0 V by operando surface‐enhanced Raman spectroscopy (SERS, Figure 1d–f).^[^ ^22^ ^]^

In general, there are two main mechanisms of electrochemical oxidation of HMF. Namely, the “electrochemical‐chemical” (E‐C) oxidation mechanism and OH*‐participated oxidation mechanism described below.

### 2.1 E‐C Mechanism

**Figure 2:**
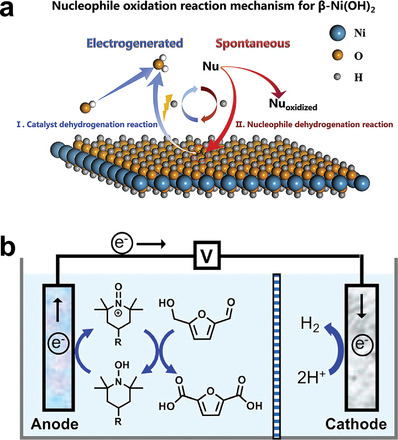
a) Scheme of nucleophile oxidation reaction mechanism for *β*‐Ni(OH)_2_. Reproduced with permission.^[^ ^23b^ ^]^ Copyright 2020, Cell Press. b) Scheme of electrooxidation of HMF by organic molecular. Reproduced with permission.^[^ ^24^ ^]^ Copyright 2019, American Chemical Society.

The E‐C mechanism involves the presence of high valence intermediate activated species. Specifically, the catalyst molecules react with OH^−^ at a certain potential to form high valence species, and then the substrate molecules are activated by high valence catalyst intermediates to obtain products, with the reduction of high valence intermediates to the initial state again. It is reported that the active site of this reaction path is electron‐deficient lattice oxygen or active adsorbed oxygen, which can activate C—H/O—H bond via hydride or hydrogen atom transfer (**Figure** **2**a).^[^ ^23^ ^]^ It should be noted that the conversion of high valence intermediates depends on the applied potential, while the subsequent HMF oxidation depends on the number of active sites or their intrinsic activity which plays a key role on the catalysts design. In addition, organic molecules are also used as oxidation media. For example, Cardiel et al.^[^ ^24^ ^]^ reported the oxidation of HMF to FDCA through the transformation of homogenous catalysts 2,2,6,6‐tetramethylpiperidinyloxyl (TEMPO) and 4‐acetamido‐TEMPO (ACT) to TEMPO^+^ and ACT^+^, respectively (Figure 2b). It is worth noting that the oxidation of homogenous catalyst only needs the transformation of electron without the participation of OH^−^, so this medium can catalyze the reaction in weak base or neutral environment.

### 2.2 OH* Mechanism

**Scheme 2:**
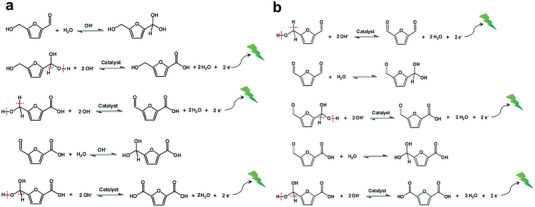
The OH* mechanisms of the electrooxidation of HMF through a) path 1 and b) path 2. Reproduced with permission.^[^ ^25^ ^]^ Copyright 2021, Royal Society of Chemistry.

The OH* mechanism does not involve the valence state transition of catalyst, and the applied voltage mainly promotes the adsorption and electron transfer of OH^−^ and subsequent substrate dehydrogenation.^[^ ^25^ ^]^ Specifically, assuming that path 1 follows this mechanism (**Scheme** **2**a). First, OH^−^ is adsorbed on the catalyst to form OH* active species at a certain potential, and then OH* activates the adsorbed HMF substrate molecule, breaking the O—H bond and C—H bond of its hydroxyl group, forming DFF. Next, water molecules attack DFF and open the C=O bond, leading to the formation of intermediate, which will be transferred to FFCA after the step of OH* activation. Finally, the product of FDCA can be obtained after repeating the water addition reaction and OH* activation. Similarly, if path 2 follows this path, it will go through five steps: HMF addition → intermediate 1 activation → HMFCA activation → FFCA addition → intermediate 2 activation, and finally FDCA is formed (Scheme 2b). The contribution of oxygen in water molecules to the reaction was confirmed by Song et al. that ^18^O‐labeled water (H_2_^18^O) was used to monitor the reaction process.^[^ ^26^ ^]^ As a result, the peaks observed from mass spectrum of oxidation products correspond to the HMFCA and FFCA with one ^18^O and FDCA containing two ^18^O, which confirms the O supply of water molecules to HMF, consistent with the OH* mechanism. However, the FDCA containing one ^18^O was also detected, so the other reaction steps cannot be ruled out. It is suggested that O in catalysts and electrolytes should also be monitored to systematically and comprehensively assess the reaction mechanism in future research. In addition, the dehydrogenation step at low voltage was confirmed by Wang et al.^[^ ^27^ ^]^ using differential electrochemical mass spectrometry, where the H* formed by deprotonation can generate H_2_ through Tafel recombination with the generation of HMFCA.

**Scheme 3:**
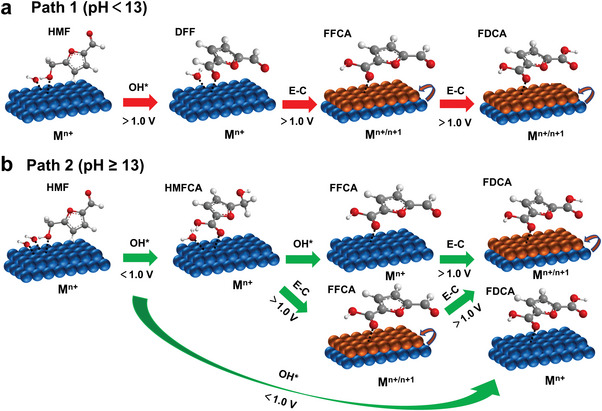
Schematic diagram of possible mixing mechanism of a) path 1 and b) path 2.

It is worth noting that the above two mechanisms are likely to coexist. Combined with the experimental results in the literature, we propose a hybrid mechanism described as follows (**Scheme** **3**).

For path 1 (Scheme 3a), the first step oxidation from HMF to DFF usually occurs in a nonstrong base environment, as mentioned above. Low OH^−^ concentrations promote the favorable adsorption of HMF due to their competitive relationship, which hinders the high valence conversion of catalyst molecules at the original potential. Furthermore, it has been proved that the formation potential of DFF is lower than the oxidation potential of catalyst.^[^ ^22^, ^28^ ^]^ The reason why high potential is applied is to promote the adsorption of OH^−^ on the electrode, so as to further activate HMF.^[^ ^25^ ^]^ Therefore, it is reasonable to assume that this step follows the OH* mechanism. The subsequent oxidation of DFF to FFCA and FDCA mainly depends on the potential and reaction time (i.e., amount of charge).^[^ ^19^, ^29^ ^]^ In essence, metal oxide catalysts will be oxidized to hydroxyl oxides under corresponding high voltage, which is considered to be the active site for the formation of FDCA.^[^ ^30^ ^]^ In addition, the high potential also promotes the deprotonation of DFF, i.e., further oxidation.^[^ ^31^ ^]^ Therefore, we believe that this step follows the E‐C oxidation mechanism. It should be noted that due to the low pH environment, this step requires higher voltage to produce FDCA than in alkaline environment, so DFF is generally served as the final product in nonstrong alkaline environment.

For path 2 (Scheme 3b), this process usually carried out in a strong alkali environment which has been analyzed above. The first step of oxidation from HMF to HMFCA can occur at a very low potential with high selectivity. Wang et al.^[^ ^27^ ^]^ synthesized HMFCA by selective oxidation of HMF at low potential by using Cu as the catalyst. Further increasing the voltage, the selectivity will be greatly reduced, which is because of the transformation of Cu to Cu_2_O, indicating that the high valence state transformation of metal catalyst is not required for the electrooxidation of HMF to HMFCA. In another study, the electrooxidation of HMF was catalyzed by Au/C to generate HMFCA, and 98% selectivity was obtained at a low potential of 0.6–0.9 V. It was confirmed that the active species of Au‐OH can be formed by Au adsorption of OH^−^ at 0.8 V, and is further oxidized to AuO*_x_* at 1.3 V.^[^ ^32^ ^]^ All the above evidences indicate that the deprotonation of HMF at low potential only requires the activated OH^−^ adsorption. Therefore, we infer that this step follows the OH* mechanism. The second step with generation of FFCA from HMFCA is complicated, because its generation potential overlaps the potential range of HMFCA and FDCA with low selectivity.^[^ ^28^, ^33^ ^]^ Therefore, this step is considered to be a mixed E‐C and OH* mechanism, which is different from path 1. It is worth noting that a small amount of FDCA is generated under low potential and high pH electrolytic environment because of the competitive mechanism of OH*.^[^ ^34^ ^]^ However, FDCA is the main product accompanied by the generation of high valence active species at high potential.^[^ ^30b^ ^]^ Therefore, it is reasonable to judge that the main mechanism of FDCA generation is E‐C. Especially, there is selective competition between FFCA and FDCA due to the similar active sites and oxidation potential, which can reach an equilibrium at a certain potential. However, the oxidation voltage range of FFCA is very narrow, so when a larger voltage is applied, the selectivity of FDCA will be greatly increased.^[^ ^35^ ^]^ If FFCA is selected as the final product, the requirement for operating voltage is strict and the selectivity seems to be low, which cannot meet the needs of elastic production in industry. Therefore, most of the current literatures take FDCA as the product of direct oxidation for HMF, whose added value is satisfactory.^[^ ^36^ ^]^

Simply, the order of redox peak and peak potential of catalysts can be detected by Linear sweep voltammetry (LSV) curve, which can help us preliminarily judge the reaction mechanism.^[^ ^23^, ^30^ ^]^ The above analysis is our reasonable conjecture on the reaction mechanism based on current experiments and theories. In addition, the trend of mechanism is also directly related to catalysts, but the structure–mechanism relationship between different catalysts is still not clearly recognized. Furthermore, there are still many mysteries to be uncovered such as the influence of interfacial water structure and the mechanism of lattice oxygen participation which have been solved in electrocatalytic water splitting.^[^ ^37^ ^]^ More detailed and accurate reaction process requires the efforts of researchers through advanced ingenious experimental means.

From the above analysis, it can be seen that the path selection of electro‐conversion of HMF is mainly related to the active site of catalyst and OH^−^ concentration. In detail, too weak substrate adsorption is not conducive to the activation and dissociation of C—H/O—H bond, while too strong substrate adsorption is not favorable to the appropriate adsorption of OH^−^ and the desorption of HMF. Therefore, an activity descriptor about substrate molecule adsorption energy was proposed by Yang et al.^[^ ^38^ ^]^ As a result, NiO has the best adsorption energy compared with other pure oxides, which is consistent with the experiment. In addition, it was also emphasized that HMF adsorption plays a decisive role under strong alkali conditions.^[^ ^38^ ^]^ However, this descriptor does not consider the transformation of active sites and the selective generation of intermediates under different bias, which is a certain gap from the real reaction. In addition, the small number of samples makes the results have certain limitations. Unfortunately, no other descriptors for this reaction have been found which need to be further explored.

## 3 Evaluating Indicator

The evaluation indexes of electrochemical conversion of HMF most concerned mainly include activity, stability, and efficiency.

### 3.1 Activity

In the laboratory electrochemical test stage, the three‐electrode system is generally carried out in the H‐type electrolytic cell. LSV can preliminarily judge the reaction activity by comparing the current density of whether there is HMF in the electrolyte. In order to obtain a specific product, the favorable potential region of the product should be selected through LSV curve, which is also related to the catalyst. Generally, the optimal voltage is always near the redox peak potential that appears before oxygen evolution reaction (OER). Then, chronoamperometry (CA) test is necessary which should be compared with chronopotentiometry (CP). However, due to the diversity of products, the product analysis depends on conversion, selectivity, yield, productivity, and Faraday efficiency (FE) which can be indirectly obtained by HPLC, and the relevant formulae are as follows

(1) $$\mathrm{Conversion}\left(\%\right)=\left(1-n_{\mathrm{HMF}-\mathrm{instant}}/n_{\mathrm{HMF}-\mathrm{initial}}\right)\times 100\%$$

(2) $$\mathrm{Selectivity}\left(\%\right)=\left(n_{\mathrm{product}}/n_{\mathrm{HMF}-\mathrm{consumed}}\right)\times 100\%$$

(3) $$\mathrm{Yield}\left(\%\right)=\left(n_{\mathrm{product}}/n_{\mathrm{HMF}-\mathrm{initial}}\right)\times 100\%$$

(4) $$\mathrm{Productivity}\left(\mathrm{mmol}\;h^{-1}\;{\mathrm{cm}}^{-2}\right)=n_{\mathrm{product}}/\left(t\times s\right)$$

(5) $$\mathrm{FE}\left(\%\right)=\left(n_{\mathrm{product}}\times n\times F/Q\right)\times 100\%$$

where *n*_HMF‐initial_, *n*_HMF‐instant_, and *n*_HMF‐consumed_ represent the initial, instant, and consumed moles of HMF, respectively, and *n*_product_ represents the moles of products. Besides, *s* corresponds to the geometric area of electrodes which also can be replaced by electrochemical active surface area, and *t* is the reaction time. In formula (5), *n*, *F*, and *Q*, respectively, represent the number of transferred electrons, Faraday constant (96 485 C mol^−1^), and total transferred charges for the generation of specific product. It is worth noting that the premise of the accuracy of the above data is *C* balance, which is easy to be ignored

(6) $$C\;\mathrm{Balance}=\left(n_{\mathrm{product}}+n_{\mathrm{HMF}-\mathrm{instant}}\right)/n_{\mathrm{HMF}-\mathrm{initial}}$$

Tafel slope can be used to judge the dynamics of HMF electro‐conversion and competitive OER. It should be noted that the reliability of Tafel slope is based on the compensation of ohmic resistance and the removal of bubble accumulation to eliminate the interference of double‐layer charging and autooxidation of catalyst.^[^ ^39^ ^]^ Although this conclusion is obtained from OER, it will also appear in HMF oxidation reaction (HMFOR). Furthermore, when the scanning rate is greater than 0.1 mV s^−1^, such interference will be intensified.^[^ ^40^ ^]^ Therefore, it is suggested to conduct electrolysis experiment at a low scanning rate. In addition, electrochemical impedance spectroscopy (EIS) can analyze mass transport and charge transfer features. Specifically, the values of the intersection of Nyquist plots with the *x*‐coordinate are related to the electrolyte impedance while the high frequency arc diameter represents the charge transfer impedance and the interface impedance, and the low frequency curve represents the mass transfer impedance.

### 3.2 Stability

**Figure 3:**
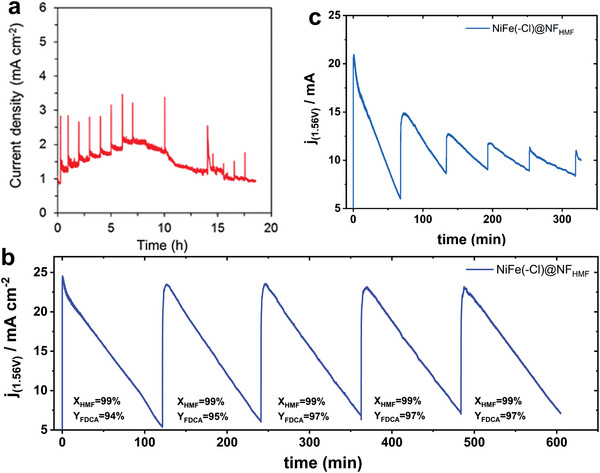
a) Current density of the HMF oxidation in the flow cell at 1.55 V versus RHE. Reproduced with permission.^[^ ^41^ ^]^ Copyright 2018, Springer. b) 10 h stability test with 5‐HMF conversion and FDCA yield for NiFe(‐Cl^−^)‐LDH@NF in 0.1 m KOH with 10 × 10^−3^ m 5‐HMF, where HPLC samples were taken and the electrolyte was replaced every 2 h; c) pulse stability test of NiFe(‐Cl^−^)‐LDH@NF. Reproduced with permission.^[^ ^42^ ^]^ Copyright 2021, Cell Press.

For the electrochemical oxidation of HMF, the amount of reactant in static electrolytic cell is limited, so the current or voltage will be minimized after long‐term test. There are three ways to solve this problem: one is to use excess amounts of HMF (**Figure** **3**a).^[^ ^41^ ^]^ However, it is not feasible in practical production, because the concentration will greatly affect other operating parameters. So, this method is only applicable to the evaluation of the long‐term stability of the catalyst in the laboratory stage. Another is to repeat the electrolytic operation after electrochemical conversion of all reactants, and compare the conversion rate, selectivity, and other data for each time (Figure 3b).^[^ ^42^ ^]^ The last method is to inject HMF regularly (Figure 3c),^[^ ^42^ ^]^ which is the most promising one we considered to be implemented in the factory. It is conducive for stable production by cooperating with the control system to keep the reaction concentration within a certain range. Since there are few studies on stability currently, the key parameters should be proposed for their standardized evaluation.

### 3.3 Efficiency

Energy consumption and energy efficiency are common indicators to measure the energy‐saving level of the system. Since most studies take hydrogen evolution reaction (HER) as the cathode pairing reaction, the energy consumption (*W*, kWh m^−3^) is calculated with the hydrogen volume as the reference value^[^ ^27^ ^]^

(7) $$W=\left(n\times F\times U\times 1000\right)/\left(3600\times V_{m}\right)$$

where *U* is the applied voltage and *V*_m_ is the molar volume of gas at normal pressure and temperature (22.4 mol L^−1^).

However, in the actual production process, the cathode pairing reaction may be diverse, so we can calculate with the mass of the main product as the reference value

(8) $$W=\left(n\times F\times U\times 1000\right)/\left(3600\times m_{\mathrm{product}}\right)$$

where *m*_product_ is the mass of the main product.

When the electrochemical conversion of HMF is carried out on both sides of the electrolytic cell, energy efficiency (*η*) can be calculated by the following formulae^[^ ^43^ ^]^

(9) $$\eta =\left|E_{\mathrm{cell}}\right|/U\times 200\%$$

(10) $$E_{\mathrm{cell}}={\mathrm{FE}}_{\mathrm{anode}}\times E_{\mathrm{anode}}-{\mathrm{FE}}_{\mathrm{cathode}}\times E_{\mathrm{cathode}}$$

(11) $$E=\Delta G^{0}/-n\;F-0.059\times \mathrm{pH}$$

where Δ*G* ^0^ (kJ mol^−1^) is the Gibbs free energy. It is noted that the maximum combined FE is 200% while the half‐reaction for the above formula is 100%.

## 4 Operating Conditions

In order to improve the activity and selectivity of the reaction, a large number of parameters have been studied. In this section, we mainly discuss the factors affecting the reaction activity and product selectivity, including reaction temperature, reaction atmosphere, reactant concentration, applied potential, substrates of working electrode, and electrolyte related factors.

### 4.1 Reaction Temperature

Reaction temperature has always been an important factor concerned by researchers, which directly affects the reaction kinetics. Zhang et al.^[^ ^31b^ ^]^ investigated the effect of reaction temperature on the electrooxidation of HMF to FDCA. However, the oxidation of HMF to FDCA is an exothermic reaction. Although the increased temperature is conducive to accelerating the reaction rate, it is not conducive to the forward reaction.^[^ ^44^ ^]^ In addition, water splitting is an endothermic reaction, and the increase of temperature will greatly promote this competitive reaction, so there is an optimal temperature for the oxidation of HMF. Unfortunately, most articles so far do not address this issue which is often the most important consideration in industrialization. In particular, the optimal temperature of different reaction systems is often different, depending on the concentration of reactants, electrolytes, and other factors. The same is true of reaction time which is directly related to the FE of the system. In the actual production process, the temperature of the whole device should be kept at the optimal anode temperature as far as possible in order to achieve higher conversion and product selectivity.

### 4.2 Reaction Atmosphere

In the traditional thermochemical oxidation of HMF, high oxygen pressure is often required,^[^ ^45^ ^]^ and the reaction atmosphere also needs to be considered in electrocatalysis which has been ignored in the current research. In addition, due to the active chemical properties of HMF, protective gas also needs to be considered in industry. Zhou et al.^[^ ^46^ ^]^ found that nitrogen or oxygen had little effect on the electrochemical test results. In order to have a fair evaluation of the reaction results, we suggest passing saturated nitrogen atmosphere in the system before the reaction, because this will not only ensure the stability of the reaction system, but also have no impact on the competitive OER.

### 4.3 Reactant Concentration

Reactant concentration is also an important factor affecting the electrochemical oxidation of HMF. The results showed that with the increase of reactant concentration, the peak current density measured by LSV increased, showing improved activity,^[^ ^30a^ ^]^ which is consistent with the results of other literatures.^[^ ^47^ ^]^ More importantly, there is a linear relationship between HMF concentration and the corresponding peak current density, indicating that the electrooxidation of HMF is a diffusion controlled process.^[^ ^30a^ ^]^ Further electrochemical impedance tests revealed the reduced charge transfer impedance between the catalyst and HMF owing to the increased concentration, so as to improve the reaction activity.^[^ ^30a^ ^]^ The first‐order kinetics models of electrooxidation of HMF were carried out for different concentrations by Liu et al,^[^ ^47a^ ^]^ and it was found that the reaction kinetics slowed down with the increase of HMF concentration. At the same time, the conversion of HMF, the selectivity of FDCA, and the FE of the reaction decreased.^[^ ^47a^ ^]^ Therefore, it can be inferred that there is an optimal reactant concentration at a certain potential for electrooxidation of HMF. It should be noted that the increase of HMF concentration always leading to the formation of humins, which could display a negative effect on the catalytic activity and makes it difficult to separate from FDCA.^[^ ^24^, ^44^, ^48^ ^]^ The above cases enlighten that each concentration has its corresponding optimized reaction potential, time, and temperature in industrial production, which should be adjusted in time to maximize the economic benefits of the whole system.

### 4.4 Applied Potential

The applied potential directly affects the coverage of intermediate and the reconstruction of catalysts in the reaction, thus leading to the formation of various products. In general, HMFCA is mainly formed at low potential (≤0.4 V), and FDCA is the main product at high potential (≥1.0 V) under alkaline conditions (pH ≥ 13).^[^ ^19^, ^27^, ^49^ ^]^ Therefore, there is an optimized potential to maximize the efficiency of the system. The same is true for the generation of DFF, accompanied by by‐product FFCA rather than FDCA,^[^ ^29^ ^]^ due to the unfavorable adsorption of HMF and OH^−^ under neutral conditions.

In addition, low potential always resulting in low productivity, which is difficult to meet industrial demand, while competitive reaction will exist and energy consumption will increase under high potential. Therefore, it is necessary to weigh the selectivity, conversion, FE, and productivity to optimize the process conditions and obtain better industrial benefits.

### 4.5 Substrates of Working Electrode

At present, foam electrode, carbon paper, glassy carbon electrode, and conductive glass are widely used as substrates of working electrode in the laboratory stage. In order to obtain reliable comparative data, the substrate should be inert to the reaction. Surprisingly, the hydrophilic enhanced carbon paper treated with sulfuric acid showed significantly increasing reactivity, which could provide inspiration for industrial development.^[^ ^46^ ^]^ Besides, Cu‐sheet was proved to be a good potential catalyst for HMF oxidation but cannot be applied as an inert support.^[^ ^50^ ^]^ It is worth noting that the reactive inertia of conductive substrate is not considered in industry, but its structural stability should be paid attention to. Here, we suggest to select the appropriate catalyst substrate based on the following two points: one is the physicochemical stability of 3D conductive substrate and the other is selectivity for the oxidation and hydrogenation of H_2_O and HMF. Besides, the industrialization cost and scalability also need to be considered. Taking the current water‐splitting device as a reference, it is recommended to use Ti felt in acidic environment, and Ni carrier electrode is often used in alkaline environment.^[^ ^51^ ^]^ However, Ni electrode has certain activity to water splitting, while Cu is inert to OER and HER, which is a good candidate. In addition, NF was not recommended to be the catalyst support in undivided or H‐type cell for HMF oxidation due to the cracking.^[^ ^46^ ^]^ Therefore, more basic research needs to be done to screen the carrier suitable for HMF oxidation.

### 4.6 Electrolyte‐Related Factors

#### 4.6.1 Electrolyte Concentration

As discussed in the second part, the acid/base environment directly determines the reaction path and product selectivity. Furthermore, the concentration of electrolyte will also affect the reaction direction. The results showed that with the electrolyte concentration increasing from 0.1 m KOH to 2 m KOH, the LSV curve gradually shifted negatively and showed improved activity,^[^ ^52^ ^]^ which may be due to the increased conductivity, faster reactant contact, and the appropriate reduction of aldehyde groups to HMF.^[^ ^53^ ^]^ Zhou et al.^[^ ^46^ ^]^ suggested that 0.1 m KOH is appropriate for the oxidation of HMF, although the optimal pH is 14, because the degradation of HMF was serious in strong alkali environment. Nevertheless, these studies did not specifically analyze the product distribution of the reaction at different pH. Another study showed that the maximum value of FE, yield, and production rate can be reached in 1 m KOH, which might be due to electrochemical and nonelectrochemical losses of HMF to unwanted side products at higher electrolyte concentration.^[^ ^50^ ^]^

However, the situation is different for electrooxidation of HMF to DFF.^[^ ^29^ ^]^ The selectivity and yield of DFF increased significantly with the decreasing pH which can be attributed to the stable aldehyde group of nonhydrate form by the resonance with aromatic nucleus under mild alkaline or neutral conditions.^[^ ^54^ ^]^ In conclusion, the concentration of OH^−^ not only affects the reaction rate, but also influence the selective oxidation of HMF. Operando SERS further confirmed that the change of OH^−^ concentration induced the surface structure transformation of the catalysts to regulate reaction products.^[^ ^22^ ^]^ In detail, alcohol groups preferentially interact with the catalyst surface and oxidize to aldehyde groups at a certain potential to form stable DFF under low pH; at high pH, aldehyde groups are preferentially oxidized under low potential, and then FDCA will be formed through the catalysis of hydroxyl oxide transformed in situ at high potential.

#### 4.6.2 Impurity Ions

The impurity ions in the electrolyte also play an important role in the electro‐conversion of HMF. The effect of cation species in electrolyte on HMF oxidation was studied by Gouda et al.^[^ ^55^ ^]^ It was displayed that the OER activity slightly improved with the increase of alkali ion radius, which was on the contrary for HMFOR. This difference was most obvious in the presence of Fe ion, because trace Fe would increase the OER activity.^[^ ^56^ ^]^ Another study emphasized the positive effect of Cu^2+^ on HMF oxidation and hydrogenation.^[^ ^46^ ^]^ In addition, Ni^2+^ and Co^2+^ have positive effects on both OER and HMFOR which should be excluded in order to avoid the loss of FE.^[^ ^57^ ^]^ The further in situ electrochemical Raman spectroscopy revealed that the origin of impurity effect was attributed to the formation of hydroxide during electrochemical measurement. In conclusion, the presence of Fe^3+^, Fe^2+^, Co^2+^, and Ni^2+^ should be avoided in the electrochemical conversion reaction of HMF and the introduction of an appropriate amount of Cu^2+^ can increase the oxidation and hydrogenation activity of HMF. In addition, attention should be paid to the corrosion and passivation of the impurity ions on the membrane and reaction device in the actual production.^[^ ^58^ ^]^

In short, the adjustment of process parameters is an important part of industrialization, and each process parameter is closely related. Researchers need to obtain the optimal conditions through theory and experiment according to the actual working conditions, so as to finally achieve the goal of improving process efficiency and saving process cost.

## 5 Catalysts Designed for Selective Production

According to the analysis of the above parts, the selective electrooxidation of HMF to FDCA, HMFCA, and DFF is relatively controllable. The catalysts for these three reactions are introduced here. However, the controllable synthesis of FFCA from HMF is still difficult, because it is often associated with the formation of other products, with low selectivity (≤60%), which has been analyzed in the part of mechanism.

### 5.1 Electrochemical Oxidation of HMF to DFF

The electrosynthesis of DFF usually requires the participation of noble metals which possess the properties of oxidation resistance and unique electronic structure. The detailed catalysts are listed in **Table** **1**.

**Table 1:** The performance of catalysts for electrochemical oxidation of HMF to DFF and HMFCA

Route	Catalysts	Electrolyte/HMF concentration [× 10^−3^ m]	Reaction time [h]	*E* (V vs RHE)	Yield/selectivity [%]	FE [%]	Ref.
HMF ↓ DFF	Pt	H_2_SO_4_(pH = 1)/20	150^b)^	2.00	16.3/16.3	‐	[59a]
	MnO*_x_*	H_2_SO_4_(pH = 1)/20	100^b)^	2.00	41.9/43.7	‐	[59a]
	Pt^a)^	0.1 m H_2_SO_4_/100	17.0	‐	≈9.5/≈73.0	‐	[59c]
	Ru^a)^	0.1 m H_2_SO_4_/100	17.0	‐	≈1.6/≈33.0	‐	[59c]
	PtRu^a)^	0.1 m H_2_SO_4_/100	17.0	‐	22.2/89.0	‐	[59c]
	Pt	0.1 × 10^−3^ m NaOH/5	12.0	2.10	18.0/25.7	‐	[59b]
	Pt/Fe_3_O_4_/rGO	0.1 m K_2_SO_4_/25	20^b)^	0.60^c)^	6.8/94.4	‐	[31a]
	Ru‐NiO	1.0 m PBS/50	10^b)^	1.50	≈18/90.0	70.0	[28]
	Co_8_Ce_2_O*_x_*	0.1 m Na_2_B_4_O_7_/5	3.0	1.50	‐/92.0	48.7	[29]
	4‐AcNH‐TEMPO	0.2 m KI, 0.5 m NaHCO_3_, 0.4 m Na_2_SO_4_/50	2.6	80^d)^	64.0/69.0	‐	[65]
	TEMPO	0.5 × 10^−3^ m LiClO_4_/0.25	20.0	1^e)^	78.0/100.0	‐	[66]
HMF ↓ HMFCA	Au	0.5 m phosphate buffer/10	6.0	1.10	50.6/92.0	84.0	[41]
	Au/C^f)^	0.1 m KOH/20	1.0	0.60	98.0/98.0	‐	[33]
	Au14	1 m KOH/5	2.0	0.82	23.6/56.9	45.8	[34]
	Pd14	1 m KOH/5	2.0	0.82	≈2.5/15.6	85.8	[34]
	Pd/C^f)^	0.1 m KOH/20	1.0	0.90	67.9/70.0	‐	[33]
	Cu	1 m KOH/50	0.5	0.40	≈70.0/100	100	[27]
	Ru‐NiO	1 m KOH/50	10^b)^	1.30	≈5.0/74.0	‐	[28]
	CoOx	0.1 m KOH/5	3	1.60	‐/48.0	‐	[29]

^a)^ Fuel cell configuration

^b)^ Passing charges (C)

^c)^ V versus Ag/AgCl

^d)^ Current density (mA cm^−2^)

^e)^ Current (mA)

^f)^ AEM‐electrolysis flow cell.

**Figure 4:**
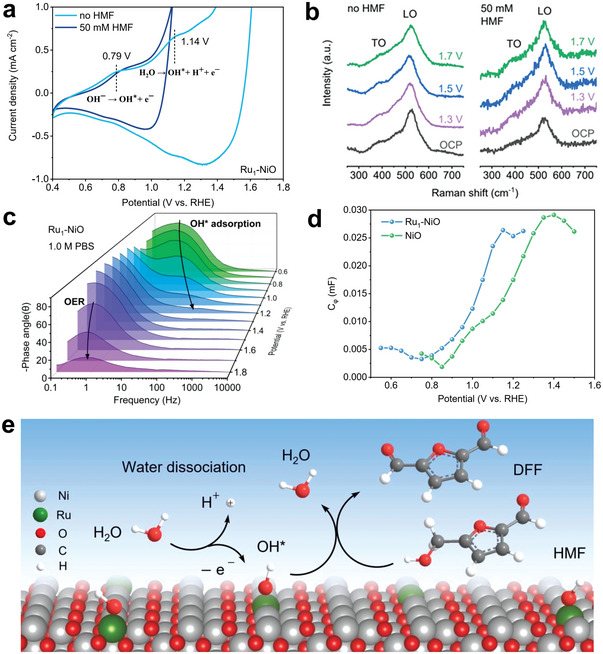
a) CV curves of Ru‐NiO in 1.0 m PBS with and without 50 × 10^−3^ m HMF; b) the ex situ Raman spectra of Ru‐NiO after electrocatalysis of 5 min; c) bode plots of Ru‐NiO which can indicate the adsorption of OH* on the catalyst surface; d) plots of C*ϕ* versus potential for catalysts in 1.0 m PBS which represent the surface coverages of OH* at the interface between Ru‐NiO and the DDL; e) proposed HMFOR mechanism over Ru‐NiO in the neutral medium. Reproduced with permission.^[^ ^28^ ^]^ Copyright 2022, Wiley‐VCH.

Among the noble metals, Pt was first tried as the catalyst for the oxidation of HMF to DFF but with low yield.^[^ ^59^ ^]^ Although a series of measures have been taken to improve its performance, the activity and stability of Pt based catalysts are always not satisfactory.^[^ ^41^, ^60^ ^]^ In addition, the catalytic selectivity of Au/C is still controversial. On the one hand, the analysis of operando Raman spectroscopy indicated that DFF is the main product of HMF oxidation with Au/C catalyst in alkaline electrolyte (pH = 12) at potential higher than 0.6 V;^[^ ^61^ ^]^ on the other hand, Au/C is considered to be an excellent catalyst for the formation of HMFCA which will be discussed in the next section. In terms of cost, the price of Ru is about 5% of Pt, and Ru is expected to become the substitute for Pt due to the similar strength of hydrogen bond.^[^ ^62^ ^]^ Although Ru black cannot efficiently convert HMF to DFF, the formation of PtRu alloy (1:1) greatly improved the HMF conversion and DFF selectivity.^[^ ^59c^ ^]^ Surprisingly, the DFF selectivity as high as 90% with 70% FE was achieved at 1.5 V in the neutral medium using single‐atom Ru on NiO as the catalyst, whose performance is the best one so far.^[^ ^28^ ^]^ Further CV, ex situ Raman spectroscopy, and operando EIS measurements indicate that the conversion of HMF to DFF under neutral electrolyte in this study follows the OH* mechanism and confirms the key role of Ru to promote adsorption and dissociation of water (**Figure** **4**).^[^ ^28^ ^]^

Nonnoble metals are also used as catalysts for the oxidation of HMF. In view of the stability of MnO*_x_* in acidic solution, 41.9% yield of DFF and 95.8% HMF conversion were achieved under acidic conditions (pH = 1) by using MnO*_x_*. However, a high voltage was required (2.0 V), leading to the formation of by‐products and a low efficiency of the system. Le et al.^[^ ^29^ ^]^ found that Co_8_Ce_2_O*_x_* had a good catalytic activity for HMF oxidation and remarkable selectively toward DFF (92%) under neutral conditions at 1.5 V for 3 h but with low FE (48.7%). X‐ray photoelectron spectroscopy (XPS) and operando Raman spectra confirmed that the incorporation of Ce has an electronic regulation effect on Co.^[^ ^29^ ^]^ Specifically, the decrease of Co^3+^/Co^2+^ ratio is a favorable factor hindering the formation of CoOOH under neutral conditions, which is the active site for generating FDCA.

In conclusion, Pt, Ru, and Pd can promote the conversion of HMF to DFF, regardless of acidic and alkaline environment, but the efficiency is unsatisfactory which may be due to the following two reasons. First, theoretically, Pt has weak adsorption on OH* and strong adsorption on H*,^[^ ^63^ ^]^ which is not conducive to the reaction. While Ru has strong adsorption on both of OH* and H*, which is more suitable in neutral environment and conducive to the dissociation of water.^[^ ^64^ ^]^ Therefore, it is necessary to consider the adsorption of active species in different ionic environments to design remarkable catalysts; second, from the perspective of operation, some of the above literature do not optimize various operating conditions, which is mentioned in the fourth part. Encouragingly, the electronic structure and surface property can be effectively regulated through loading, alloying, doping, and other ways, so as to improve the activity and even stability. Besides, considering the corrosive effect of acidic electrolyte on catalyst and electrolytic cell components, it is recommended to operate in neutral environment, where the aldehyde group is stable. It should be noted that the adsorption and dissociation steps of water need to be considered to provide active OH*, which can refer to the excellent catalysts for alkaline HER. In addition, the formation of high valence oxidation active species should be avoided to prevent further oxidation of DFF.

### 5.2 Electrochemical Oxidation of HMF to HMFCA

As mentioned above, alkaline environment is conducive to the formation of HMFCA which is different from that of DFF. For noble metals, Au is considered to be the best catalyst for the oxidation of HMF to HMFCA.^[^ ^33^, ^34^, ^41^ ^]^ While using Pd/C as catalyst, Chadderdon et al.^[^ ^33^ ^]^ believed that HMFCA was mainly formed at high potential (0.9–1.2 V), and FFCA was generated through DFF intermediate at low potential (0.6 V). Therefore, Pd/C is not recommended for the formation of HMFCA. For nonnoble metal, the production of single product HMFCA was realized at ultra‐low voltage (0.4 V) by Cu catalyst, which can also be applied to diverse range of aromatic aldehydes.^[^ ^27^ ^]^ Interestingly, Cu^0^ → Cu^1+^ was observed when the potential reached 0.5 V, which inhibited the formation of HMFCA.^[^ ^27^ ^]^ Therefore, it is important to compare the redox potential with different valence states of catalysts and the oxidation potential of reactants.

In addition, metal oxides are also used as catalysts for HMF oxidation to HMFCA. NiO supported Ru atom obtained about 74% selectivity of HMFCA at 1.3 V under alkaline conditions but low yield.^[^ ^28^ ^]^ Although the mechanism has not been analyzed, we can infer from its characterization that the incorporation of high valence Ru into NiO lattice results in the decrease of valence state of Ni, which inhibits the formation of high reactive species in the electrochemical oxidation process and avoids the further formation of FDCA. CoO*_x_* was also served as catalyst for the oxidation of HMF that 48% selectivity and considerable yield of HMFCA were obtained at 1.6 V.^[^ ^29^ ^]^ Further research on CoO*_x_* H*_y_* shows that selective HMFCA production is mediated by Co^2+^/Co^3+^ redox couple.^[^ ^29^ ^]^

To sum up, there are two main types of catalysts used for HMFCA production: one is the metallic catalyst following OH* mechanism at low potential, and the other is the metallic oxide catalyst through low valence transition following E‐C mechanism at high potential (Table 1). It is worth noting that the second type is not the mainstream mechanism for HMFCA production which has a low yield. Although a high selectivity can be obtained, it is likely to be the illusion caused by non‐faradaic process.^[^ ^67^ ^]^ For example, 96% selectivity was achieved using CeO*_x_* as catalyst, while the current density turned out to be 0 in the *i*–*t* curve, although at a high voltage of 1.6 V.^[^ ^29^ ^]^ Therefore, the comparative experiments should be done to eliminate the influence of non‐faradaic reaction, which has been seriously ignored. For the catalyst design of HMFCA, on the one hand, antioxidant metals or metals with higher redox potential should be selected to avoid the generation of high valence oxides in the alkaline environment, which is conducive to the further oxidation of HMFCA. On the other hand, different electron configurations of the same valence state also result in different selectivity, which requires further study. Finally, it is recommended to use simple metal as the catalyst due to the high selectivity toward HMFCA with high catalytic activity at a low voltage (≤1 V), resulting in less energy consumption.

### 5.3 Electrochemical Oxidation of HMF to FDCA

HMFCA is often used as an intermediate during the production of FDCA under alkaline conditions at higher potentials, because aldehydes are not stable in alkaline media, which will react quickly in the presence of oxygen or other electron acceptors.^[^ ^68^ ^]^ Since FDCA is the most popular production in the electrochemical conversion of HMF, the types of catalysts are subdivided in order to better discuss the active sites, which are also listed in **Table** **2**.

**Table 2:** Performance of different catalysts with derived active sites for conversion of HMF to FDCA

Categories	Catalysts	Active sites	Electrolyte/HMF concentration [× 10^−3^ m]	Reaction time [h]	*E* (V vs RHE)	Yield/selectivity [%]	FE [%]	Ref.
Metals and alloys	Pd_1_Au_2_/C	‐	0.1 m KOH/20	1.0	0.90	83.0/83.0	‐	[33]
	Pd_7_/Au_7_	‐	1.0 m KOH/5	2.0	0.82	16.4/38.7	85.8	[34]
	Ni NPs	Ni^3+^	0.1 m KOH/10	‐	‐	‐	‐	[21b]
	Ni/CP	NiO/NiOOH	0.1 m KOH/5	44^b)^	1.36	99.4/99.7	‐	[69]
	Cu foam	Hydroxide and oxide	0.1 m KOH/5	41^b)^	1.62	96.4/96.4	95.3	[70]
	NiCu NTs	‐	1.0 m KOH/20	2.0	1.424	99.0/99.0	96.0	[103]
	FeSn_2_	*α*‐FeO(OH)	1.0 m KOH/20	2.0	10^c)^	‐	90.0	[72]
	FeSi	Oxidic iron(III)	1.0 m KOH/100	1.5	20^c)^	‐	94.0	[73]
Oxides	NiO‐CMK‐1	NiOOH	0.2 m KOH/20	1.5	1.85	51.4/79.0	70.0	[35a]
	Co‐NiO	NiO(OH)_ads_	1.0 m KOH/10	87^b)^	1.47	94.8/97.2	94.6	[38]
	NiO‐Co_3_O_4_	NiOOH	1.0 m KOH/10	60^b)^	1.45	98.0/98.0	96.0	[20b]
	Co_3_O_4_	Co^3+^ and Co^3+/4+^	1.0 m KOH/50	‐	1.50	≈85.0/‐	≈78.0	[78]
	CuCo_2_O_4_	Co^3+^_oh_	1.0 m KOH/50	≈62^b)^	1.45	93.7/‐	94.0	[76]
	Ir‐Co_3_O_4_	‐	1.0 m KOH/50	≈58^b)^	1.42	98.0/‐	98.0	[79a]
	Vo‐Co_3_O_4_	‐	1.0 m KOH/10	≈58^b)^	1.47	91.9/‐	88.1	[79b]
	NiCo_2_O_4_	Co^3+^	1.0 m KOH/5	≈0.9	1.50	90.4/90.8	≈94.0	[30c]
	NiCo_2_O_4_	NiOOH	1.0 m KOH/10	190^b)^	1.45	99.0/99.4	99.0	[104]
	Ni_0.5_Co_2.5_O_4_	‐	1.0 m KOH/50	60^b)^	1.50	92.4/‐	90.4	[78]
	(FeCrCoNiCu)_3_O_4_	‐	1.0 m KOH/10	60^b)^	1.50	≈98.0	≈97.0	[82a]
	CuMn_2_O_4_	Mn^4+^‐O	1.0 m KOH/10	6.0	1.31	‐	98.4	[82b]
Hydroxides	NiO/Ni(OH)_2_	‐	1.0 m NaOH/5	4.0	16^c)^	71.0	84.0	[84]
	Pt‐Ni(OH)_2_	Ni(OH)O	1.0 m KOH/50	‐	‐	‐	‐	[85]
	CoOxHy	Co^3+^ and Co^3+/4+^	1.0 m KOH/10	≈58^b)^	1.50	‐	70.0	[86]
	Cu(OH)_2_	CuOOH	1.0 m KOH/100	6.0	0.8^d)^	98.7/‐	100.0	[30a]
	NiFe LDH	‐	1.0 m KOH/10	10.0	1.23	98.0/99.0	99.4	[47a]
	d‐NiFe LDH	MOOH	1.0 m KOH/10	5.0	1.48	96.8/99.4	84.5	[87]
	CoAl LDH	‐	0.1 m KOH/10	59.1^b)^	1.52	‐	99.4	[26]
	NiCoFe LDH	‐	1.0 m NaOH/10	1.0	1.52	84.9/88.9	≈90.0	[31b]
	NiCoMn LDH	NiOOH and Mn^2+/3+^	1.0 m NaOH/1	2.5	1.50	91.7/91.7	≈65.0	[44]
	CoFe@NiFe LDH	Ni^3+^	1.0 m KOH/10	3.5	1.40	100.0/100.0	99.8	[49]
	Cu*_x_* S@NiCo LDH	Ni^3+^	1.0 m KOH/10	58^b)^	1.32	99.0/99.0	99.0	[105]
Hydroxyl oxides	NiOOH	Ni(OH)_2_/NiOOH	0.1 m KOH/5	4.7	1.47	96.0/96.2	96.0	[30b]
	CoOOH	Co(OH)_2_/CoOOH	0.1 m KOH/5	22.0	1.56	35.1/36.8	35.1	[30b]
	FeOOH	FeOOH	0.1 m KOH/5	2.3	1.71	1.6/10.0	1.6	[30b]
MOFs and COFs	t‐NiCo‐MOF	Ni^2+/3+^	1.0 m KOH/50	57.8^b)^	‐	100.0/100.0	98.0	[91]
	NiCoBDC‐NF	Ni with high valence	0.1 m KOH/10	4.0	1.55	99.0/‐	78.8	[89]
	CoNiFe‐MOFs/NF	Oxyhydroxide	1.0 m KOH/10	1.7	1.40	99.8/‐	100.0	[90]
	TpBpy‐Ni@FTO	‐	0.1 m LiClO_4_/0.5	2.9^b)^	1.55	58.0/60.4	‐	[92]
Borides	NiB*_x_*	Ni^3+^	1.0 m KOH/10	≈1.7	0.60^e)^	98.8/99.0	99.5	[97]
	Ni*_x_* B^a)^	Oxidized Ni species	1.0 m KOH/10	0.5	1.45	98.5/98.5	100.0	[21a]
Nitrides	Ni_3_N@C	NiOOH	1.0 m KOH/10	174^b)^	1.45	98.0/98.0	99.0	[20a]
	Ni_3_N	Ni^2+*δ*^N(OH)_ads_	1.0 m KOH/50	≈58^b)^	1.47	≈92.0/≈92.0	‐	[19b]
	Ni_3_N‐V_2_O_3_	Ni^3+^	1.0 m KOH/10	1.87	‐	96.1/98.7	‐	[101a]
	VN	‐	1.0 m KOH/10	≈0.9	20^f)^	94.1/96.0	84.0	[98]
Phosphides	Co‐P/CF	‐	1.0 m KOH/50	6.0	1.423	90.0/90.0	‐	[35b]
	Ni_2_P NPA/NF	Oxidized Ni species	1.0 m KOH/10	2.5	1.423	100.0/100.0	98.0	[95a]
	NiFeP	Ni(OH)_2_‐NiOOH	1.0 m KOH/10	86.8^b)^	1.435	99.4/99.4	94.6	[96]
	NiCoP	NiOOH	1.0 m KOH/10	57.9^b)^	1.50	‐	87.2	[106]
	MoO_2_‐FeP	‐	1.0 m KOH/10	2.7	1.42	98.0/98.6	97.8	[47b]
	NiP‐Al_2_O_3_/NF	‐	1.0 m KOH/0.3	3.5	1.45	97.8/99.6	‐	[101c]
Sulfides	Ni_3_S_2_/NF	‐	1.0 m KOH/10	58^b)^	1.423	98.0/98.0	98.0	[95b]
	Co_0.4_NiS@NF	NiCo(O)OH	1.0 m KOH/10	57.8^b)^	1.45	>99.0/>99.0	99.1	[107]
	N‐MoO_2_‐Ni_3_S_2_	‐	1.0 m KOH/10	‐	1.623	90.0/100.0	‐	[101b]
	Co_9_S_8_‐Ni_3_S_2_@NSOC/NF	‐	1.0 m KOH/10	116^b)^	1.40	98.8/98.8	98.6	[108]
	NiS_x_/Ni_2_P	Ni^3+^	1.0 m KOH/10	90^b)^	1.46	98.5/98.5	95.1	[19a]
Selenides	NiSe@NiO*_x_*	NiO*_x_*	1.0 m KOH/10	≈2	1.423	99.0/99.0	99.0	[109]
	CoO‐CoSe	OVs‐CoO	1.0 m KOH/10	≈1	1.43	99.0/99.0	97.9	[100]
	NF@Mo‐Ni_0.85_Se	‐	1.0 m KOH/10	60^b)^	1.40	95.0/95.0	95.0	[99]

^a)^ Flow cell

^b)^ Passing charges

^c)^ Constant current density (mA cm^−2^)

^d)^ V versus Ag/AgCl

^e)^ V versus standard hydrogen electrode

^f)^ Constant current (mA).

#### 5.3.1 Metals and Alloys

**Scheme 4:**
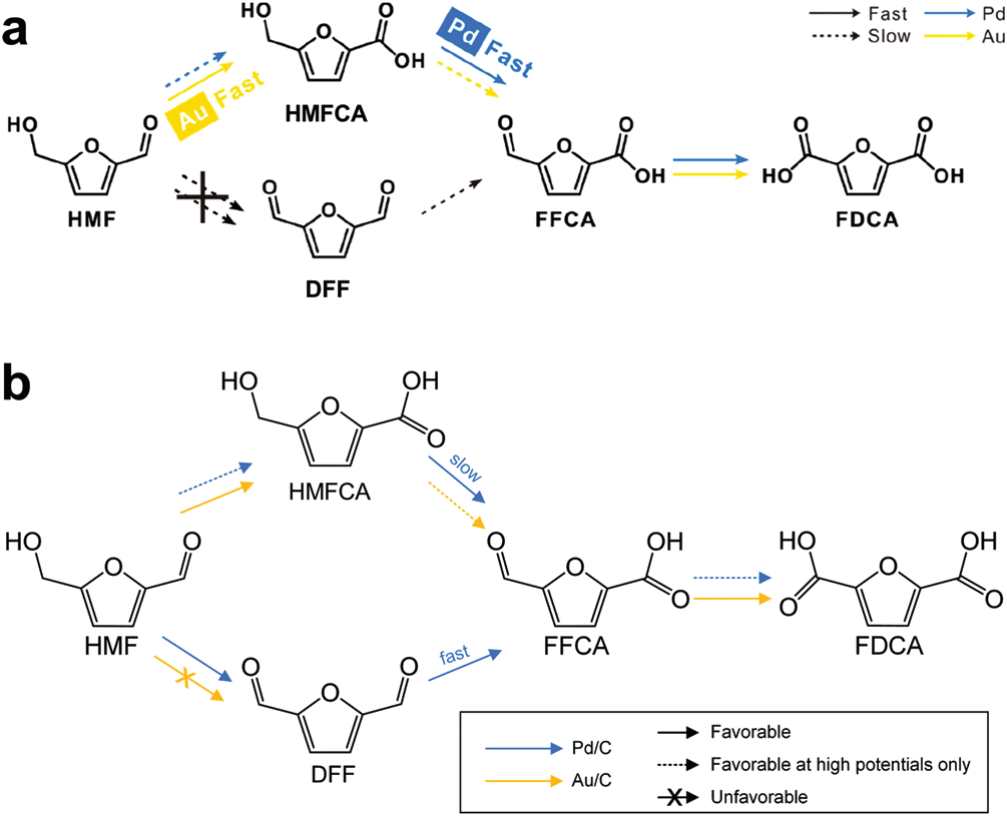
a,b) Controversial reaction pathways of HMF oxidation on Pd/C and Au/C electrocatalysts in alkaline media. a) Reproduced with permission.^[^ ^34^ ^]^ Copyright 2020, American Chemical Society. b) Reproduced with permission.^[^ ^33^ ^]^ Copyright 2014, Royal Society of Chemistry.

The role of noble metals in the conversion of HMF to HMFCA and DFF has been mentioned above, while single metals are not efficient enough to oxidize HMF to FDCA. Therefore, considering the synergy of alloys, Chadderdon et al.^[^ ^33^ ^]^ confirmed the high activity of Pd_1_Au_2_/C, with 83% selectivity and 100% HMF conversion under only 0.9 V. Further surface composition analysis revealed the existence of pure Au phase, which can improve the activity of separated Pd.^[^ ^33^ ^]^ Finally, aldehyde and hydroxyl groups were activated by Au and Pd, respectively, jointly converting HMF into FDCA. Similar work was carried out by Park et al.^[^ ^34^ ^]^ that the one with rich Au on outer layer (Pd_7_/Au_7_) has superior selectivity than that of Pd (Au_7_/Pd_7_). However, although the alloying elements are the same in the above two studies, the preferred path of forming FDCA is different (**Scheme** **4**). According to the controversial research results, it may be due to the different morphology and electronic structure resulting from different preparation methods which lead to surface exposure to different crystal faces. As a result, the differential adsorption energy under electrochemical state finally affects the selectivity. Therefore, the structure–activity relationship of catalysts needs further study. In addition, we encourage to judge in the same system with unified standards, because there are great differences between flow cell and half‐reaction cell. Although noble metals show considerable activity, their post reaction characterization is insufficient, and no further active site identification is put forward.

As for nonnoble metals, a high voltage is usually required to generate high valence active species through indirect oxidation of HMF. Morphology engineering was performed to effectively improve the activity by Poerwoprajitno et al.^[^ ^21b^ ^]^ In detail, faceted branched nickel nanoparticles are prepared, which is beneficial for the exposure of high active crystal surface, promoting favorable HMF adsorption and H—O bond dissociation. As a result, active nickel species with the valence of +3 can be obtained at a low potential, facilitating the formation of FDCA. Similarly, the in situ formed Ni^2+^ and Ni*^*δ*^* ^+^ (*δ* = 2–3) from Ni^0^ were found in the form of NiO or NiOOH, served as the reason for the enhanced activity of carbon paper‐supported Ni nanosheet.^[^ ^69^ ^]^ Besides, nanocrystalline Cu foam shows superior performance than bulk Cu, which is attributed to the high content of amorphous hydroxide and oxide formed on the surface.^[^ ^70^ ^]^

**Figure 5:**
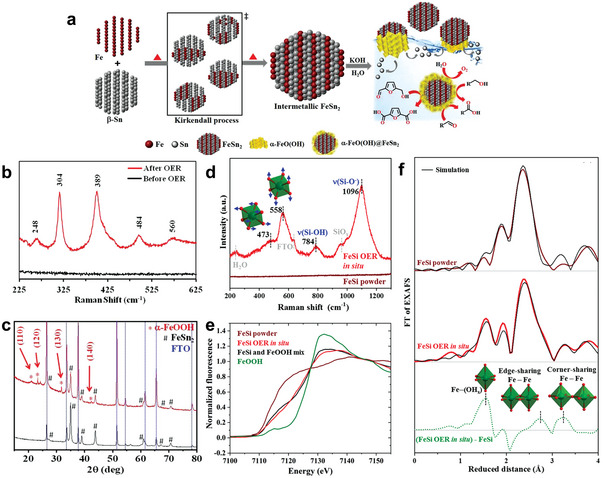
a) Schematic representation of the formation of FeSn_2_ and *α*‐FeIIIO(OH)@FeSn_2_ and b) Raman spectra of the FeSn_2_/FTO before (black) and after OER CA (red), c) PXRD pattern of the FeSn_2_/FTO before (black) and after OER CA (red). Reproduced with permission.^[^ ^72^ ^]^ Copyright 2020, Wiley‐VCH. d) Raman data of pristine FeSi powder and in situ during OER. The numbers represent the frequencies in cm^−1^ of the Raman bands and e) Fe K*α* XANES spectra. The black line is a linear combination of FeSi powder and FeOOH in a 1 to 1 ratio, f) EXAFS spectra together with their simulations. The green dotted line is the result of the subtraction of the FeSi powder spectrum FT amplitude from the FeSi OER in situ spectrum FT amplitude to gain an EXAFS plot that reflects the newly formed iron(III) phase. Reproduced with permission.^[^ ^73^ ^]^ Copyright 2021, Wiley‐VCH.

In addition, as a kind of alloy, intermetallics possess unique electronic structure and crystal structure which has special advantages in electrocatalysis.^[^ ^71^ ^]^ Taking FeSn_2_ as the conductive core, the in situ formed *α*‐FeO(OH) on the surface by OER activation was confirmed by Raman spectra and powder X‐ray diffraction (PXRD, **Figure** **5**a–c), which exhibited 90% FE to produce FDCA.^[^ ^72^ ^]^ Besides, Hausmann et al.^[^ ^73^ ^]^ also confirmed that the in situ formed oxidic iron(III) on FeSi was the active phase to catalyze the conversion of HMF to FDCA with FE of 94 ± 3%. Moreover, the iron(III) phase consisting of edge and corner sharing [FeO_6_] octahedra was further proved by Raman, X‐ray absorption near edge structure (XANES), and extended X‐ray absorption fine structure (EXAFS) spectra (Figure 5d–f). However, which kind of structure is the essential active center remains to be studied. It is worth noting that in previous studies, it has been confirmed that edge‐shared octahedron is the active center of OER due to its more favorable OH^−^ adsorption than corner‐shared octahedron.^[^ ^74^ ^]^ In both cases, the surface active oxidation species activated by OER are used as HMF oxidation catalysts. Although they have good activity, which may lead to competitive reaction at high potential. Therefore, further active site regulation is needed to expand the active potential window of HMF oxidation in future research.

From the above results, it can be inferred that the in situ‐derived hydroxide or hydroxyl oxide is the active species to produce FDCA. Nevertheless, there is still a long way to go for metals and alloys to obtain FDCA where active high valence metal oxide is usually involved, urged by high voltage.

#### 5.3.2 Metal Oxide, Hydroxide, Hydroxyl Oxide

**Figure 6:**
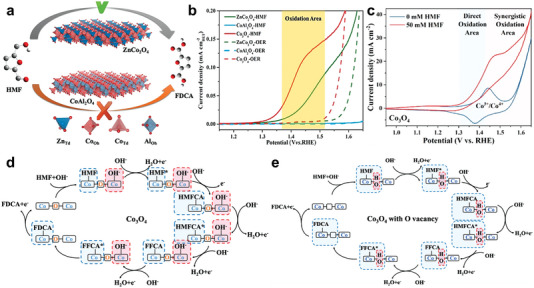
a) A Scheme to show the relative performance for electrochemical HMF oxidation to the targeted product on spinel oxides by building geometric sites of the tetrahedron (Zn^2+^) or octahedron (Al^3+^) and b) LSV curves of ZnCo_2_O_4_, Co_3_O_4_, and CoAl_2_O_4_ normalized by BET area. Reproduced with permission.^[^ ^76^ ^]^ Copyright 2020, Wiley‐VCH. c) Cyclic voltammograms of Co_3_O_4_ in 1 m KOH at a scan rate of 50 mV s^−1^. Reproduced with permission.^[^ ^78^ ^]^ Copyright 2022, American Chemical Society. d,e) The reaction mechanism of HMFOR on Co_3_O_4_ and Vo‐Co_3_O_4_. Reproduced with permission.^[^ ^79b^ ^]^ Copyright 2021, Wiley‐VCH.

Metal oxides have great advantages in oxidation reactions due to the flexibility in chemical compositions and electronic states.^[^ ^75^ ^]^ At present, the Ni‐based and Co‐based oxide are the most studied owing to their excellent adsorption properties.^[^ ^38^ ^]^ For NiO, the related researches mainly focus on the surface structure modification and electronic structure regulation.^[^ ^35^, ^38^ ^]^ Further research revealed that NiO(OH)_ads_ or NiOOH was the active center.^[^ ^20^, ^38^ ^]^ For Co_3_O_4_, Wang's group^[^ ^76^ ^]^ revealed the role of different geometric configurations of cobalt in Co_3_O_4_. In particular, Co^2+^ at tetrahedral sites (Co^2+^_Td_) can chemically adsorb acidic organic molecules, while octahedral Co^3+^ (Co^3+^_Oh_) plays a decisive catalytic role in the oxidation of HMF (**Figure** **6**a,b). It is worth noting that Co^3+^_Oh_ sites are also the best geometrical configuration for OER.^[^ ^77^ ^]^ Therefore, the geometry regulation of catalytic sites is worthy of further study. Then, it was confirmed that the catalytic process of Co_3_O_4_ for HMF oxidation contained both the direct oxidation of Co^3+^ and the indirect oxidation of Co^3+/4+^ (Figure 6c).^[^ ^78^ ^]^ On the other hand, the regulation of adsorption behavior in Co_3_O_4_ was further explored.^[^ ^79^ ^]^ Specifically, due to the introduction of oxygen vacancy (Vo) in Co_3_O_4_, OH^−^ can fill into Vo in the lattice of Vo‐Co_3_O_4_. The involvement of lattice OH^−^ not only breaks the competitive adsorption between OH^−^ and HMF, but also accelerates the rate determining step of the hydrogenation of HMFCA through lattice oxygen oxidation reaction process (Figure 6d,e).^[^ ^79b^ ^]^ Furthermore, the adsorption energy of Co_3_O_4_ can be regulated by monatomic Ir, resulting in strengthened adsorption of C=C group in HMF.^[^ ^79a^ ^]^ Moreover, other methods such as morphology engineering can also improve the performance due to more exposed active sites.^[^ ^52^, ^80^ ^]^

Thanks to the special antispinel structure of NiCo_2_O_4_ that nickel ion occupies the octahedral position and cobalt ion occupies both the octahedral and tetrahedral position, an excellent activity better than Co_3_O_4_ and NiO is obtained.^[^ ^30^, ^81^ ^]^ However, the active center is still uncertain that Kang et al.^[^ ^30c^ ^]^ identified Co as the active site, which is contrary to the view of Gao et al.^[^ ^81^ ^]^ Recently, Lu et al.^[^ ^78^ ^]^ found that Co_3_O_4_ has higher activity for aldehyde group than hydroxyl group, while NiO has high activity for hydroxyl group. Then, the hydroxyl activity of Co_3_O_4_ is regulated by Ni doping at tetrahedral sites, so as to improve the oxidation performance of HMF.^[^ ^78^ ^]^ It is worth noting that Ni doping also changes the reaction mechanism to direct oxidation. Other oxides such as high‐entropy oxide and Mn‐based spinel are also good catalysts, but the researches are still in the initial stage.^[^ ^82^ ^]^ In general, metal oxide catalysts need to be further enriched, not limited to Ni‐ and Co‐based, such as perovskite oxides due to its flexible and adjustable structure. In addition, the methods to improve the performance of oxides need to be developed, such as the construction of heterojunction, which can improve the adsorption of reactants and the electron transfer during reaction.^[^ ^83^ ^]^

**Figure 7:**
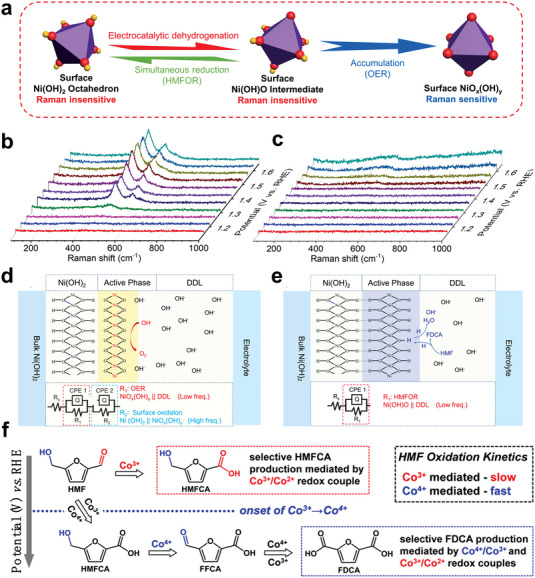
a) Schematic representation of crystal structure transformation among surface Ni(OH)_2_ octahedron, surface Ni(OH)O intermediate, and complex surface NiO*_x_* (OH)*_y_*, b) operando Raman spectroscopy during OER and c) HMFOR, d) schematic illustrations of OER and e) HMFOR system at Ni(OH)_2_ electrode. Reproduced with permission.^[^ ^85^ ^]^ Copyright 2021, Wiley‐VCH. f) Mechanistic illustration of the potential‐dependent oxidation of HMF to produce HMFCA and FDCA, as mediated by electrogenerated Co^3+^ and Co^4+^ species. Reproduced with permission.^[^ ^86^ ^]^ Copyright 2021, Wiley‐VCH.

The use of metal hydroxide catalysts can be traced back to 1991.^[^ ^84^ ^]^ It is the first successful case of electrocatalytic oxidation of HMF to FDCA, where 71% yield was obtained by NiO/Ni(OH)_2_.^[^ ^84^ ^]^ Zhou et al.^[^ ^85^ ^]^ adjusted the adsorption energy of Ni(OH)_2_ with HMF through the introduction of Pt, and explored the transformation of active species Ni*^*δ*^* ^+^ in the process of HMFOR. Especially, ex situ XPS spectra and operando Raman spectra confirmed that Ni(OH)_2_ is electrooxidized to Ni(OH)O, which further oxidized HMF to FDCA without forming NiO*_x_* (OH)*_y_*, considered as the active species of OER (**Figure** **7**a–e).^[^ ^85^ ^]^ For CoO*_x_* H*_y_*, different from the mechanism of Co_3_O_4_, it was confirmed that Co^3+^ plays the role of oxidizing aldehyde group to form HMFCA, and the redox pair of Co^3+/4+^ is the key species to form FDCA (Figure 7f).^[^ ^86^ ^]^ So, we can reduce the formation barrier of Co^4+^ by means of high electronegativity or low‐cost element doping, etc., to improve the efficiency. Other hydroxides, such as Cu(OH)_2_, whose active site is CuOOH, have recently been used as HMF oxidation catalysts which exhibited high activity with FE close to 100% and 98.7% yield of FDCA.^[^ ^30a^ ^]^

In recent years, as a kind of hydroxide, a large number of layered double hydroxide (LDH) catalysts have emerged for HMF oxidation. The first case was carried out by Liu et al.^[^ ^47a^ ^]^ through NiFe‐LDH, where 99.4% FE was achieved better than Ni(OH)_2_. The subsequent researches of LDHs mainly focused on regulating the electronic structure to inhibit competitive reaction of OER. For example, Song et al.^[^ ^26^ ^]^ prepared self‐supporting ultra‐thin CoAl‐LDH by in situ electrochemical stripping, resulting in rich oxygen vacancies and improved adsorption of HMF. In addition to oxygen vacancies, the role of metal defects has also been revealed by Qi et al.^[^ ^87^ ^]^ In detail, the existence of cationic defect (M^2+^) in NiFe‐LDH moved up the d‐band center which resulted in improved electronic structure, and the authors further attributed the active species to MOOH.^[^ ^87^ ^]^ However, the effect of defects on the reaction mechanism and the formation of active sites has not been deeply studied.

**Figure 8:**
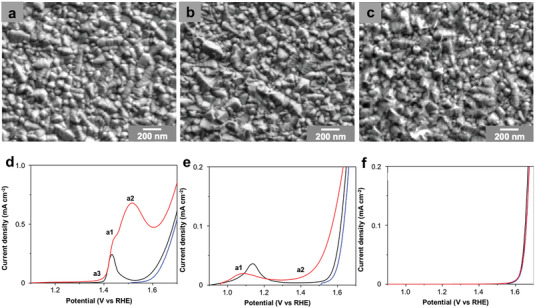
SEM images of thin a) NiOOH, b) CoOOH, c) FeOOH films used for voltammetric studies. LSVs of thin d) Ni(OH)_2_, e) Co(OH)_2_, and f) FeOOH films in a 0.1 m KOH solution (scan rate: 5 mV s^−1^). Three consecutive LSVs were obtained in the same solution; the first (black) and second (blue) LSVs were performed in the absence of HMF, while the third (red) was performed after the addition of 5 × 10^−3^ m HMF. Reproduced with permission.^[^ ^30b^ ^]^ Copyright 2018, American Chemical Society.

In view of the possible active center of hydroxyl oxide in the above researches, MOOH (M = Fe, Co, Ni) films were directly prepared by Taitt et al.^[^ ^30b^ ^]^ to study the mechanism (**Figure** **8**a–c). In detail, LSV indicated that the activity order of the MOOH follows NiOOH > CoOOH > FeOOH (Figure 8d–f), in which NiOOH and CoOOH may follow both direct oxidation and indirect oxidation steps (a1 and a3 regions in Figure 8d,e), and the result is consistent with what we described in the mechanism part. However, the existence of a2 region in NiOOH is not found and explained in other researches until Bender et al.^[^ ^23a^ ^]^ served it as Ni^2+^/Ni^4+^, belong to potential‐dependent indirect oxidation of HMF. This is still unknown that why Ni‐based catalysts exhibit similar active sites but different active behaviors, which may be related to the local coordination environment, requiring further exploration.

#### 5.3.3 MOF, COF‐Based Catalysts

Metal‐organic frameworks (MOFs) and covalent‐organic frameworks (COFs) have recently been used as catalysts for HMF oxidation due to their highly porous structure and adjustable electronic structure of metal sites.^[^ ^88^ ^]^ Cai et al.^[^ ^89^ ^]^ verified the feasibility of MOF for HMF oxidation for the first time. In particular, 2D NiCo‐MOFs showed the best activity with a high FDCA yield of 99% at 1.55 V (pH = 13). Benefiting from the coupling with Co and terephthalic acid ligand modification, high valence Ni species was stable which are considered as the catalytic sites. Similar conclusions were confirmed in FeCoNi ternary MOFs by Bai et al.^[^ ^90^ ^]^ that the electron withdrawing Fe and Co promoted the formation of high valence Ni species. In addition to directly used as a catalyst, MOFs can also act as a pre‐catalyst. Deng et al.^[^ ^91^ ^]^ converted NiCo‐MOF to NiCo‐LDH through CV activation, which showed unprecedented HMF catalytic activity that 600 mA cm^−2^ can be obtained at 1.4 V with a high FE of 98%. The authors attribute the high activity to the electronic interaction between nickel and cobalt, leading to the generation of high valence transition metal cations formed under a lower oxidation potential. Using COFs as the catalyst for FDCA generation was first demonstrated with nickel(II)‐doped TpBpy‐Ni film immobilized on fluorine‐doped tin oxide (FTO) by Cai et al.^[^ ^92^ ^]^ However, only 58% FDCA yield was obtained with 96% HMF conversion, possibly due to the low surface electroactive Ni coverage.

Therefore, it is generally believed that the high valence species are the active site for oxidation of HMF to FDCA, following the E‐C mechanism. It can be seen from the above cases that such catalysts show amazing catalytic activity especially for MOFs, but further exploration is necessary, such as the structure–activity relationship and in situ transformation process during the reaction. In addition, the stability of MOFs in acid/alkali environment has been criticized, which is mainly affected by the alkalinity of organic structural units, the acidity of metal oxide clusters, and other factors.^[^ ^93^ ^]^ COFs also have some problems in molecular stacking and biocompatibility. Moreover, as a new frame structure, metal‐covalent organic frameworks (MCOFs) are expected to achieve a balance in crystallinity, porosity, and stability, deserving further study.^[^ ^94^ ^]^

#### 5.3.4 Metal Borides, Nitrides, Phosphides, Sulfides, and Selenides

**Figure 9:**
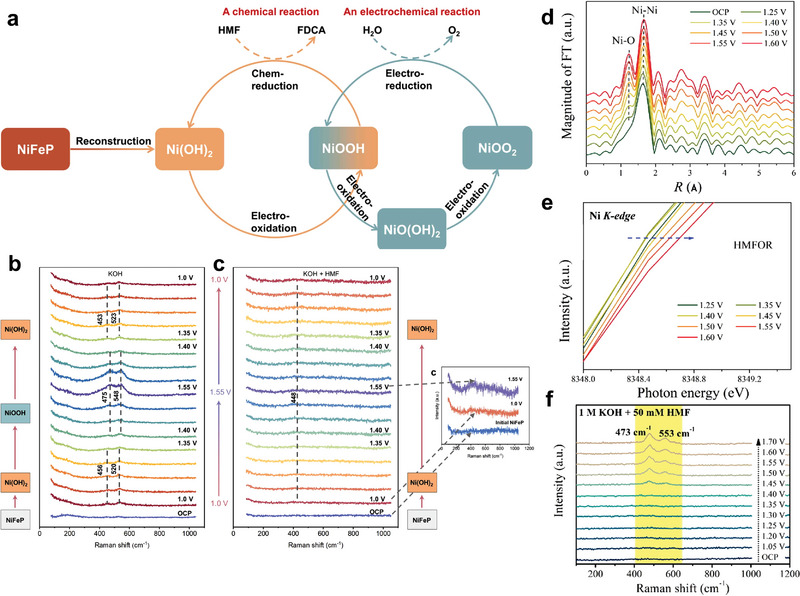
a) Schematic diagram of the oxyhydroxide‐centered dual‐cycle mechanism for competitive OER and HMFOR and in situ Raman study of NiFeP catalysts during b) OER and c) HMFOR in 0.1 m KOH electrolyte with 10 × 10^−3^ m HMF. Reproduced with permission.^[^ ^96^ ^]^ Copyright 2022, Elsevier. d) Fourier transformed EXAFS spectra of Ni_3_N during HMFOR and e) in situ XANES spectra of Ni K‐edge, f) in situ Raman spectra of Ni_3_N for HMFOR. Reproduced with permission.^[^ ^19b^ ^]^ Copyright 2021, Elsevier.

The use of metal phosphides and sulfides for HMF oxidation was first studied by Sun's group.^[^ ^35^, ^95^ ^]^ In these cases, HMF was transformed efficiently with ≥90% yield of FDCA in alkaline media. It is worth noting that the post‐electrolysis analysis revealed the partial disappearance of P/S and the oxidation of Ni/Co which are considered to be active species in HMF oxidation. Recently, the process of surface reconstruction was studied with NiFeP as catalyst by Luo et al (**Figure** **9**a).^[^ ^96^ ^]^ Further in situ Raman revealed the reversible cyclic catalytic oxidation of HMF dominated by Ni(OH)_2_ and NiOOH (Figure 9b,c).^[^ ^96^ ^]^ Similarly, as for borides, Zhang et al. and Brawe et al.^[^ ^21^, ^97^ ^]^ confirmed that the oxidation species of Ni^+3^ formed on the surface of NiB*_x_* during the catalytic process is the active center. Furthermore, Zhou et al.^[^ ^19b^ ^]^ studied the active sites of Ni_3_N during HMF oxidation by in situ XANES and EXAFS spectra (Figure 9d,e). As a result, with the increasing applied voltage, the adsorbed Ni^2+^ *^*δ*^* N(OH)_ads_ species increase gradually (Figure 9d) with higher valence of Ni (III) (Figure 9e), served as the active sites to promote the oxidation of HMF, which was further proved by in situ Raman (Figure 9f).^[^ ^19b^ ^]^ However, Zhang et al.^[^ ^20a^ ^]^ identified the +3 valence species of Ni_3_N after HMFOR as NiOOH to be the active site. In addition, VN also showed excellent HMF oxidation performance with FDCA selectivity ≥96% and ≥84% FE.^[^ ^98^ ^]^ Further density functional theory reveals that the closer d‐band center to the Fermi level leads to better HMF adsorption.^[^ ^98^ ^]^ Interestingly, Yang et al.^[^ ^99^ ^]^ downshift the d‐band center of Ni in Ni_0.85_Se by Mo doping, resulting in reduced H* adsorption energy which can promote the dehydrogenation of organic molecules. The above cases indicate that the d‐band center of active metal should be adjusted to the optimal position to balance HMF adsorption and dehydrogenation.

At present, the modification of catalysts mainly focuses on the construction of heterojunction, which can realize effective regulation of electronic structure. For example, the NiS*_x_*/Ni_2_P not only improved the adsorption of HMF than NiS*_x_*, but also promoted hole accumulation in Ni_2_P and the formation of high valence Ni in NiS*_x_*, which jointly boosted the oxidation of HMF.^[^ ^19a^ ^]^ Besides, CoO/CoSe_2_ heterojunction improved the reaction activity by introducing abundant oxygen vacancies.^[^ ^100^ ^]^ In addition, the electron transfer between heterojunctions makes it possible for simultaneous oxidation of HMF and hydrogen production.^[^ ^101^ ^]^ Yang et al.^[^ ^47b^ ^]^ constructed MoO_2_‐FeP heterojunction and confirmed the electron transfer from MoO_2_ to FeP at the interface. Especially, the electron accumulation on FeP optimized the absorption energy of H_2_O and H*, while the hole accumulation on MoO_2_ contributes to improved HMFOR activity.

Based on the above different types of catalyst cases, the active site and reaction mechanism of HMF oxidation to FDCA can be summarized as follows: 1) indirect oxidation at medium potential through M^2+/3+^; 2) the coexist of direct oxidation at low potential and indirect oxidation at medium potential; 3) in addition to the above processes, the direct or indirect oxidation at high potential through M^3+/4+^ may exist. In fact, the above phenomena have long been found in OER,^[^ ^102^ ^]^ and the apparent difference is that the HMF molecule can be adsorbed and activated with active site at a lower potential. Since the generation voltage of FDCA is similar to that of OER, the deeper difference between OER and HMFOR deserves further study, including the reconstruction of catalysts, the difference of active sites (whether lattice oxygen is involved or not), the difference of active valence and geometric configuration, so as to broaden the potential range of FDCA production and improve the FE relative to OER.

### 5.4 Photoelectrochemical (PEC) Oxidation of HMF

**Scheme 5:**
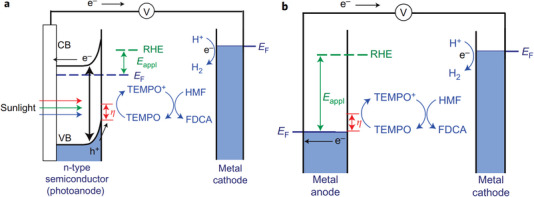
Comparison of the PEC and electrochemical cells. a) PEC TEMPO‐mediated HMF oxidation. b) Electrochemical TEMPO‐mediated HMF oxidation. CB, conduction band; EF, Fermi energy. Reproduced with permission.^[^ ^110^ ^]^ Copyright 2015, Springer Nature.

PEC catalytic oxidation is an effective and sustainable scheme to reduce the cost of electrocatalysis and improve the efficiency of photocatalysis. However, there are few reports on PEC oxidation of HMF where photogenerated holes are responsible for the oxidation of redox media, and the applied bias only enhances the electron–hole separation which is different from the process of electrochemical HMF oxidation (**Scheme** **5**). Choi and Cha first confirmed the feasibility of PEC oxidation of HMF to FDCA at PEC anode in 2015.^[^ ^110^ ^]^ Specifically, FDCA with nearly 100% yield was obtained at 1.04 V after passing of 40 C.^[^ ^110^ ^]^ The following research mainly focused on the improvement of BiVO_4_ photoanode or the exploration of new photoanode.^[^ ^111^ ^]^

The future work should focus on developing more effective and cheaper photoanodes with smaller band gaps, and designing more appropriate batteries with minimum internal resistance drop,^[^ ^112^ ^]^ so as to minimize input of electricity while ensuring higher total energy efficiency. In addition, homogenous oxidized media need to be further screened to improve conversion efficiency.

## 6 Design of Reactors

The configuration of electrolytic cell is an important segment which must be considered in industrial continuous production. At present, only one industrial level project has been put into production with HMF oxidation to FDCA, where TEMPO is used,^[^ ^36^ ^]^ and there is no systematic study on reactor for electrochemical conversion of HMF. Therefore, the insights into practical‐scale electrochemical conversion of HMF are urgently needed.

### 6.1 Classification of Reactors

#### 6.1.1 Undivided Cell and H‐Type Cell

**Figure 10:**
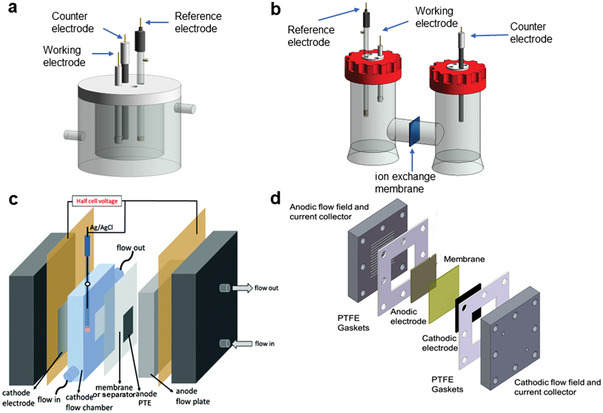
Schematic illustration of a) undivided cell, b) H‐type cell, c) flow cell. Reproduced with permission.^[^ ^43^ ^]^ Copyright 2021, Royal Society of Chemistry, and d) MEA‐based electrolyzer. Reproduced with permission.^[^ ^42^ ^]^ Copyright 2021, Cell Press.

Undivided cell (**Figure** **10**a) is the simplest type of electrolyzer where ion exchange membrane is not required. However, the liquid products on both sides are likely to react with each other, which reduce the overall efficiency and increase the separation cost.^[^ ^66^, ^101^ ^]^ At present, most studies on the electrochemical conversion of HMF remain in H‐type cell (Figure 10b) where the potential of cathode or anode can be accurately controlled and the interaction of reactions on both sides can be avoided. However, only static analysis and simple evaluation can be conducted in H‐type cell, which is far from continuous production in actual industry.

#### 6.1.2 Flow Cell

In view of the above view, the research about flow cell (Figure 10c) is necessary which can strengthen mass transfer and reduce ohmic loss of the system, resulting in reduced energy consumption. In addition, the reference electrode can be used to accurately control the voltage of half‐cell, which provides a basis for simulating the dynamic process of actual production. Liu et al.^[^ ^43^ ^]^ conducted paired electrolysis of HMF reduction to DHMF and oxidation mediated by TEMPO. Then, the electrolytic activity of H‐type and flow cell was compared, which indicated that the energy efficiency of the flow cell is increased by more than four times compared to the H‐type cell due to the reduced resistance.^[^ ^43^ ^]^ In addition, for the operating conditions of the flow cell, we cannot fully refer to the H‐type cell, due to different system impedance, mass transfer, and reaction residence time, which need to be regulated by flow rate, liquid supply mode, and other parameters in the actual production process.^[^ ^113^ ^]^

The advantages of membrane‐free flow cell (Figure 10c, replacing ion exchange membrane with separator) include faster build time, less maintenance, longer service life, simple operation, and design scalability. Recently, Li et al.^[^ ^32^ ^]^ realized electrochemical oxidation of benzyl alcohol coupled with cathodic hydrogen production with high current in a membrane‐free flow cell, although the current density is still low (160 mA cm^−2^), which is also one of the industrial challenges of electrochemical conversion of HMF. Zhu et al.^[^ ^114^ ^]^ separated the two electrodes with plastic mesh to construct a membrane‐free continuous flow cell. Finally, 0.92 g FDCA powder was obtained at the average reaction rate of 5.22 mmol_FDCA_ g^−1^_cat_ h^−1^ by using DHMF as the reactant.

However, the fetal disadvantages of flow cell are the large ohmic loss and ion imbalance, resulting in reduced production efficiency, which needs further improvement. Therefore, the most promising mainstream for industrial amplification is zero‐gap MEA‐based electrolyzer which has developed rapidly in water splitting.^[^ ^115^ ^]^ It should be noted that the selection of reactor must consider the paired reaction. For example, if the cathodic coupling reaction is a gas reduction reaction, a gas diffusion electrode with additional gas channels should be used, which has been described in detail in another review.^[^ ^116^ ^]^ This paper mainly expounds the system with liquid reactant.

#### 6.1.3 MEA‐Type Electrolyzer

MEA‐based electrolyzer (Figure 10d) is generally composed of bipolar plates (BPPs), current collectors, porous transport layer (PTL), catalyst layer (CL), and ion exchange membrane.^[^ ^117^ ^]^ The special zero‐gap assembly has many advantages, such as lower contact impedance, faster ion transmission, and start‐up response,^[^ ^118^ ^]^ exhibiting promising industrial application potential.

**Figure 11:**
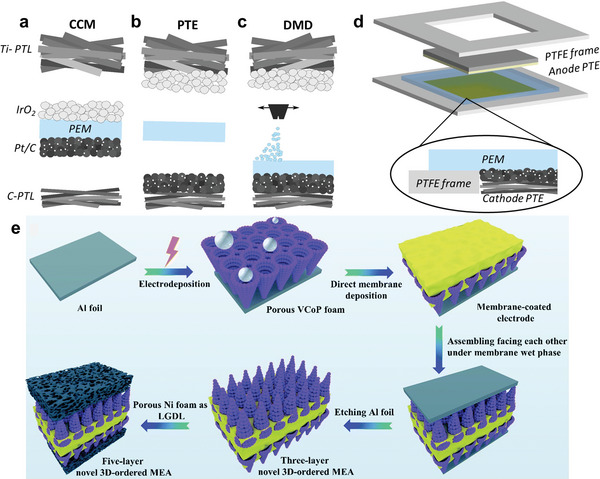
Schematics from a) state‐of‐the art CCM and b) PTE‐type MEA to c) Direcet membrane deposition MEA (DMD‐MEA); d) sealing concept based on DMD‐MEA setup membrane is directly deposited onto the cathode PTE and the PTFE frame. Reproduced with permission.^[^ ^125^ ^]^ Copyright 2019, Elsevier. e) Step synthesis of novel 3D‐ordered MEA by electrodeposition and direct membrane deposition methods. Reproduced with permission.^[^ ^126^ ^]^ Copyright 2022, Royal Society of Chemistry.

Conventionally, there are two main MEA configurations: catalyst coated membrane (CCM) electrodes (**Figure** **11**a) and porous transport layer electrodes (PTEs, Figure 11b). On the one hand, as for CCM, CL is closely connected with the membrane by spraying or decal transfer method to reduce the interface contact resistance and promote ion transport.^[^ ^119^ ^]^ For the spraying method, although less process flow and cost are required, the swelling problem of the membrane is tricky, which needs further improvement.^[^ ^120^ ^]^ While the decal transfer method shows a better interface with the membrane, the operation process needs to be simplified especially in low temperature.^[^ ^121^ ^]^

On the other hand, as for PTE, coating and sputtering are the most common methods for the construction of the PTE. Among the coating methods, air‐brushing, hand‐painting, and screen‐printing are common methods for the preparation of PTE where air‐brushing tends to exhibit a more uniform and flat distribution of CL.^[^ ^122^ ^]^ In addition, sputtering is one of the common methods for preparing thin film by physical vapor deposition, which has broad application prospects due to the accurate control of the thickness and loading of CL.^[^ ^123^ ^]^ Due to the firm connection between catalyst and PTL through chemical bonds, the stability of long‐term operation can be ensured in self‐supporting PTE, and the porous substrate can provide more exposed active sites for conversion of HMF. Moreover, ionomer is not required in self‐supporting PTE. Therefore, the promotion of self‐supporting PTE is recommended to adapt to the harsh industrial conditions.

No matter which of the above assembly methods, the membrane, CL, and PTL will eventually be integrated by hot‐pressing to ensure the best connection.^[^ ^124^ ^]^ In addition, some novel assembly methods have also been proposed. For example, Holzapfel et al.^[^ ^125^ ^]^ directly deposited the membrane on the cathode PTE, showing a smaller ohmic impedance and mass transfer impedance (Figure 11c,d). Wan et al.^[^ ^126^ ^]^ constructed a 3D‐ordered MEA by direct membrane deposition technique, and mass transfer channels were developed in CL, membrane, and the interfaces, which effectively reduced the internal resistance of the whole system (Figure 11e). Although the above results were not confirmed in the HMF conversion reaction, it provides basic guidance for the amplification of HMF conversion. The following mainly discusses the matters needing attention in the operation of MEA electrolyzer, so as to better guide the industrial amplification.

### 6.2 Membrane

**Figure 12:**
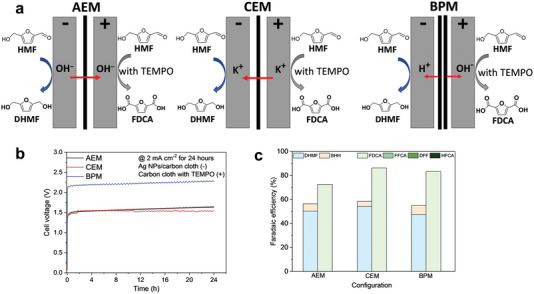
a) Schematic illustration of the MEA configurations with different ion‐selective membranes, including AEM, CEM, and BPM. Red arrows indicate the possible charge‐carrying ionic species that transport across the membranes. b) Cell voltage profiles for continuous 24 h electrolysis in the systems with AEM, CEM, and BPM at 2 mA cm^−2^ (10 mA). c) FE of electrochemical hydrogenation (left columns) and electrochemical oxidation (right columns) in different configurations. Reproduced with permission.^[^ ^128^ ^]^ Copyright 2019, Chemistry Europe.

Generally speaking, cation exchange membrane (CEM) can be used under acidic conditions, while anion exchange membrane (AEM) is used under alkaline conditions. Besides, bipolar membrane (BPM) is often applied when the pH difference of electrolyte is existent on both sides.^[^ ^127^ ^]^ Therefore, for the different HMF conversion reactions mentioned above, different membranes can be selected according to their ion transport characteristics. However, from the current literature, proton exchange membrane (PEM, a kind of CEM) is often preferred even for HMF oxidation reaction in alkaline environment.^[^ ^31^, ^43^, ^47^, ^97^, ^98^ ^]^ So, the objective evaluation of the three membranes is urgently needed. Liu et al.^[^ ^128^ ^]^ evaluated the activity of three types of membranes for electrochemical conversion of HMF in MEA electrolyzer (pH = 9.2) (**Figure** **12**a). The cell voltage profiles showed that a higher voltage was required in BPM‐MEA (Figure 12b), resulting from higher membrane resistance and the additional voltage needed for water dissociation,^[^ ^129^ ^]^ indicating higher energy consumption.^[^ ^128^ ^]^ Further product analysis showed that membrane has no effect on the reduction of HMF to DHMF in cathode, while AEM‐MEA showed the worst FE for the oxidation of HMF to FDCA in anode due to species crossover (Figure 12c).^[^ ^128^ ^]^ At present, CEM is the most mature one, while AEM and BPM developed slowly. Among them, BPM‐MEA is a novel reactor, whose excellent performance has been confirmed in water electrolysis,^[^ ^130^ ^]^ which provides a new idea for HMF conversion, such as anodic alkaline HMFOR coupling with cathodic acidic HER. For AEM, a higher efficiency for furfural electrochemical reduction in AEM‐MEA versus CEM‐MEA had been demonstrated in acid electrolyte due to limited HER, but the disintegration was observed.^[^ ^131^ ^]^ Furthermore, Hauke et al.^[^ ^42^ ^]^ explored the effects of different AEMs on the oxidation of HMF to FDCA. The results showed that FAA‐3‐PK130 exhibited the best performance possibly due to its special network structure which prevented organic species blocking the membrane channel during electrolysis.^[^ ^42^ ^]^

Based on the above views, both AEM and CEM can be used in MEA electrolyzer for HMF conversion. However, it needs to be emphasized that different reaction systems, reactant supply methods, water management should be considered to select targeted membranes and systematic research under industrial conditions is needed in the subsequent research.

In addition to the applicability of membrane, there are still some problems remain to be solved in the application. First, the issues of reactant or product crossover and membrane degradation need to be paid attention to. It has been confirmed that the crossover of HMF and its products could existence in both AEM and PEM.^[^ ^46^ ^]^ However, there is no targeted research to provide solutions. In fact, the crossover of small organic molecules has long been found in proton exchange membrane fuel cell (PEMFC), which can be used for reference.^[^ ^132^ ^]^ Second, for membrane degradation under long‐term operation, it has been found that Fe ion causes the degradation of PEM through Fenton reaction.^[^ ^58^, ^133^ ^]^ Then, Thomassen et al.^[^ ^58b^ ^]^ evaluated the degradation of PEM by online monitoring the concentration of fluorine ion at the outlet of the reactor which can be popularized as a degradation index in industry. Therefore, during the process of catalyst preparation, device assembly, and raw material transportation, the content of Fe ion should be strictly controlled which can also promote competitive OER. With regard to the degradation mechanism of different AEMs, related content is detailed in another review.^[^ ^134^ ^]^

In addition, the appropriate amount of ionomer is of great significance for the whole performance. Specifically, lower ionomer content leads to poor ion conduction and unstable CL network, leading to high ohmic impedance. While excessive ionomer content hinders the electron transport of the catalyst and causes high mass transfer impedance.^[^ ^116^ ^]^ The optimum ionomer content should be determined according to the catalyst load, distribution, and other factors, which needs to be further adjusted in practical operation.^[^ ^135^ ^]^

### 6.3 PTL

Although the HMF conversion process does not involve gas production, PTL is still necessary because of its favorable mass transfer channel.^[^ ^136^ ^]^ The selection of PTL material should be based on different acid/base environment. For alkaline or neutral environment, metal foam substrates are recommendable, such as Ni foam and Cu foam which have been successfully applied in HMFOR.^[^ ^27^, ^42^ ^]^ Under acidic environment, corrosion is the biggest barrier for PTL, especially under high voltage and current. Currently, most studies use Ti fiber as anode PTL in acidic condition. However, under long‐term operation, a passivation layer will be formed, which hinders electron transfer leading to increased ohmic impedance and reduced efficiency.^[^ ^137^ ^]^ Therefore, lots of researches are devoted to the preparation of anticorrosion coatings, including noble metals (Pt, Au, Ir)^[^ ^137^, ^138^ ^]^ and nonnoble metal (NbN)^[^ ^139^ ^]^ coatings. It is worth noting that the PTL material is less stringent for the cathode under the reduction current, and just the cheap carbon paper can meet the demand.^[^ ^140^ ^]^ However, carbon‐based PTL is not recommended to be used on the anode side due to possible oxidation under harsh working conditions, resulting in the collapse of catalyst.

The structural properties of PTL closely related to the utilization of catalyst, interfacial contact impedance, mass and charge transfer, thus affecting the cell performance.^[^ ^141^ ^]^ Various parameters such as thickness, porosity, and pore gradient have been studied in water splitting,^[^ ^142^ ^]^ which provides a certain guiding significance for the electrochemical conversion of HMF. In addition, the properties that may involve side reactions need to be paid attention to, such as the hydrophobicity and wettability of PTL. Besides, the segmented structure design of PTL can also regulate the reaction residence time, which was recently discovered by Zhang et al.^[^ ^143^ ^]^

Based on the above views, the next research should focus on the large‐scale preparation of structure controllable and anticorrosion PTL, especially for acidic condition. For example, Lettenmeier et al.^[^ ^144^ ^]^ produced PTL with controllable pore distribution by vacuum plasma spraying, showing great potential to reduce commercial costs. Hackemüller et al.^[^ ^145^ ^]^ scaled the dimensions of the PTL up to 47 × 47 cm^2^ by tape casting, exhibiting industrial application potential for manufacturing large‐scale PTL.

**Figure 13:**
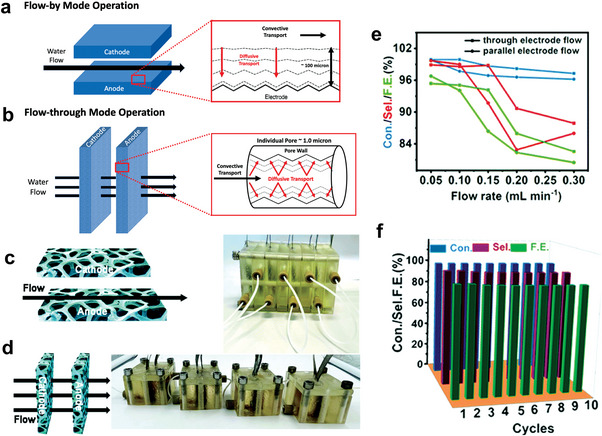
Electrochemical reactor operation in a) flow‐by mode operation and b) flow‐through mode operation. Reproduced with permission.^[^ ^146^ ^]^ Copyright 2019, American Chemical Society. The designed and 3D‐printed component assembly of the c) flow‐by reactor and d) flow‐through reactor; e) HMF conversion, FDCA selectivity, and FE under different flow rates of the flow‐through and flow‐by reactors; f) the HMFOR of the flow‐through reactor under ten successive cycles. Reproduced with permission.^[^ ^136^ ^]^ Copyright 2019, Royal Society of Chemistry.

During industrial continuous production, different flow modes between supply solution and PTL greatly affect the mass transfer of reactants, which lead to different energy consumption and production efficiency. Specifically, there are two forms of flow: flow‐by and flow‐through. In flow‐by mode, flat plate electrodes are often used in traditional electrochemical cells where a thick diffusional boundary layer about 100 µm will form, resulting in limited mass transfer (**Figure** **13**a).^[^ ^146^ ^]^ In the flow‐through mode, the PTE is often applied, where the convective transport of reactants can be realized, which reduces the thickness of the diffusion boundary layer, resulting in favorable mass transfer (Figure 13b).^[^ ^146^ ^]^ Wang et al.^[^ ^136^ ^]^ compared the two flow modes reactors applied for HMF oxidation (Figure 13c–f), and the superior activity of nearly 30 times higher than that of the flow‐by reactor was confirmed with flow‐through mode by using space‐time yields as index. This study exhibited the broad application prospect of MEA electrolyzer with flow‐through mode in electrochemical conversion of HMF.

### 6.4 BPPs

The cost of bipolar plates accounts for a major part of the MEA electrolyzer, especially the use of Ti‐BPPs under acidic conditions.^[^ ^147^ ^]^ Similar to the PTL of Ti fiber, the current researches on BPPs under acidic conditions mainly focus on improving the corrosion resistance of Ti‐BPPs and reducing its cost. Specifically, a large number of anticorrosion coatings have been tried, including Ti, Nb, Ta, and their N‐compounds.^[^ ^148^ ^]^ Recently, Ti and Nb/Ti nonnoble metal coatings were coated on stainless steel BPP and PTL, respectively, by Stiber et al.,^[^ ^149^ ^]^ and the long‐term stability under high temperature and high current was confirmed, which made it possible to fundamentally reduce the cost of Ti‐BPPs and Ti‐PTL. On the contrary, under alkaline conditions and neutral conditions, stainless steel BPPs can meet the requirements which reduce the application costs.^[^ ^42^, ^150^ ^]^

The design of flow channel in BPPs affects the diffusion behavior of fluid and reaction residence time, which is directly related to the efficiency of the system. The cell performance with different flow channels has been analyzed in fuel cell and water electrolysis, but the conclusions are inconsistent. Majasan et al.^[^ ^151^ ^]^ believed that parallel flow field promoted fluid flow, while Rho et al.^[^ ^152^ ^]^ and Arif et al.^[^ ^153^ ^]^ confirmed that the configuration of serpentine flow field was better. However, the HMF electrolysis does not involve gas production, so the situation will be different and further conclusion needs to be verified.

## 7 Summary and Prospects

Combined with advanced in situ characterization and innovative experimental methods, exciting results has been achieved in understanding the reaction mechanism and designing electrocatalyst of HMF electrolysis in the last few years. This review emphasizes the reaction mechanism of HMF electrolysis and the important details that are easy to be ignored in practice. In addition, according to the different products, the recent advances of the electrocatalysts are discussed, and finally the design of the reactor is prospected. However, there is still a certain gap between the current achievements and the industrial production which needs to be further explored in the following aspects.

### 7.1 Consistency between Reaction Mechanism and Active Site

The reaction mechanism and reaction path in the literature are still controversial, resulting in the inconsistency between reaction mechanism and active site. For example, either the formation of Ni^3+^ or Ni^4+^ is the active site of indirect oxidation of HMF, and either the metal ion or lattice oxygen is the adsorption site of HMF. The contradiction can be attributed essentially to the inability to accurately identify active sites, which rely on the development of advanced in situ characterization technology. In addition, due to the possible reconstruction phenomenon in the reaction process, the research on the reaction mechanism and active site should also focus on the following points: 1) formation reason of reconstructed active layer; 2) the difference between the active species formed by in situ reconstruction and directional synthesis; 3) how to regulate the reconstruction behavior to further improve the activity.

### 7.2 Design of Applicable Descriptors

So far, only one descriptor has been proposed and only partial oxides are applicable.^[^ ^38^ ^]^ Therefore, descriptors with wide applicability need to be established. Moreover, the quantitative relationship between descriptor and intrinsic activity of electrocatalyst is also a difficult problem to be solved. Fortunately, many descriptors have been explored for the electrochemical conversion of small molecules that can be used as a reference. For example, the d‐band center is often used as an indicator of the binding strength of intermediates in the process of electrochemical reaction,^[^ ^154^ ^]^ which is expected to be used in HMF electrolysis. In addition, the machine‐learning algorithm is considered to be a powerful tool for developing descriptors based on linking the reaction performance parameters with electrochemical reaction thermodynamics, electrode dynamics, and other parameters in the literature.^[^ ^155^ ^]^ It is worth noting that many electrocatalysts will undergo reconstruction in the reaction process, especially oxidation reaction, which adds difficulties to the establishment of descriptors due to the change of actual active sites.

### 7.3 Developing Electrocatalyst for Practical Production

At present, although most studies have achieved high HMF conversion and FE under a low current density, operation under high current density (≥500 mA cm^−2^) is necessary in industrial production for higher efficiency. However, this is extremely challenging due to the fierce competitive reaction (HER or OER). There are two possible solutions: On the one hand, the types of catalysts are not rich enough, so more catalysts remain to be tried such as perovskite oxide, high‐entropy alloy, single‐atom catalysts, and so on. In addition, modification methods need to be explored to further improve the reaction performance such as molecule modification. On the other hand, accurately identify and regulate the active sites of HMF electrolysis and its competitive reaction are urgently needed which can fundamentally realize the separation between main and side reactions. Of course, the guidance of theoretical calculation is essential to help screen catalysts, such as machine‐learning.^[^ ^156^ ^]^ In addition to the development of highly active catalysts, excellent stability of electrocatalysts is an important factor in industrial production. However, the criteria used to evaluate the stability of catalysts under working conditions have not been established. Therefore, the preparation of large‐scale electrocatalysts with high stability and activity under high current is the focus of future research.

### 7.4 Applicability of Paired Reaction

The HMF oxidation and reduction reaction described in this paper can be carried out in one reactor or paired with other reactions according to the actual needs. Currently, the reactions paired with HMF electrolysis include HER,^[^ ^95a^ ^]^ oxygen reduction reaction,^[^ ^157^ ^]^ CO_2_ reduction reaction,^[^ ^158^ ^]^ and N_2_ reduction reaction,^[^ ^159^ ^]^ exhibiting great multidirectional application potential. However, reactions paired with HMF reduction still need to be developed, such as the electrooxidation of sulfide. It is worth noting that although above paired electrolysis has broad application prospects, the adaptability needs to be further tested in practice, because the voltage of anode and cathode cannot be controlled at the same time in the two‐electrode system. Basically, the coupling reaction should possess similar dynamics and charge transfer number.^[^ ^160^ ^]^ In addition, the cost of the process needs to be evaluated to maximize production benefits.

### 7.5 Popularization of MEA Electrolyzer and Design of New Reactors

The application of MEA electrolyzer in HMF electrolysis reaction is mainly introduced in Section 6, which has not been widely used in current research, and there are still many basic problems remain to be solved. Systematic research needs to be done to comprehensively understand the influence of membrane, ionomer, CL, and BPPs on the performance of electrolytic cell. In addition, various parameters under working conditions should be explored, such as flow rate, liquid supply mode, etc. In these aspects, simulation calculation is important to reduce experimental trial and error. Moreover, the design of new reactors also needs to be tried, such as microchannel flow reactor,^[^ ^161^ ^]^ which can further narrow the electrode space and accurately guide the flow path.^[^ ^162^ ^]^ For further industrial integration and amplification, advanced cases can be used for reference, such as redox flow battery module stack, which has been carried out by the Department of Chemical Engineering at the University of Bath and in industry.^[^ ^163^ ^]^ It is worth noting that ionic liquids provide favorable conditions for the electrolysis of HMF in an acid–base free environment, which can further reduce the device costs. However, current research on ionic liquid‐assisted HMF conversion does not involve external voltage input where high temperature and noble metal catalysts are always required.^[^ ^164^ ^]^

### 7.6 Separation of Products

The product separation after HMF conversion must be considered in industrial production. At present, the method of product separation is mainly based on thermodynamic difference and solubility difference, which is a high‐cost process in industrial production. Although the production process of some products has been matured in the thermochemical conversion of HMF, the separation process may not be applicable for electrochemical production due to different reaction system and technology design. Therefore, low cost separation methods suitable for electrochemical industrial production system need to be developed, such as membrane separation technology. In addition, the use of homogenous catalysts, such as TEMPO, further increases the separation and recovery cost. Therefore, heterogenous catalysts are still the primary choice at present, because of its easy recovery and controllable structure.

## Conflict of Interest

The authors declare no conflict of interest.
